# Genome-wide association study provides novel insight into the genetic architecture of severe obesity

**DOI:** 10.1371/journal.pgen.1011842

**Published:** 2025-09-12

**Authors:** Mohanraj Krishnan, Mohammad Yaser Anwar, Anne E. Justice, Geetha Chittoor, Hung-Hsin Chen, Rashedeh Roshani, Alyssa Scartozzi, Rachel R. Dickerson, Roelof A. J. Smit, Michael H. Preuss, Nathalie Chami, Benjamin S. Hadad, Esteban J. Parra, Miguel Cruz, Qin Hui, Peter W. F. Wilson, Yan V. Sun, Xiaoyu Zhang, Gregorio V. Linchangco, Sharon L. R. Kardia, Jessica D. Faul, David R. Weir, Lawrence F. Bielak, Heather M. Highland, Kristin L. Young, Baiyu Qi, Yujie Wang, Myriam Fornage, Christopher Haiman, Iona Cheng, Ulrike Peters, Charles Kooperberg, Steven Buyske, Joseph B. McCormick, Susan P. Fisher-Hoch, Frida Lona-Durazo, Jesus Peralta, Jamie Gomez-Zamudio, Stephen S. Rich, Kendra R. Ferrier, Ethan M. Lange, Christopher R. Gignoux, Eimear E. Kenny, Genevieve L. Wojcik, Kelly Cho, Michael J. Gaziano, Luc Djousse, Shuwei Liu, Dhananjay Vaidya, Renée de Mutsert, Navya S. Josyula, Christopher R. Bauer, Wei Zhao, Ryan W. Walker, Jennifer A. Smith, Leslie A. Lange, Mariah C. Meyer, Ching-Ti Liu, Lisa R. Yanek, Miryoung Lee, Laura M. Raffield, Ruth J. F. Loos, Penny Gordon-Larsen, Jennifer E. Below, Kari E. North, Mariaelisa Graff

**Affiliations:** 1 Department of Epidemiology University of North Carolina at Chapel Hill Gillings School of Global Public Health, Chapel Hill, North Carolina, United States of America; 2 Carolina Population Center, University of North Carolina at Chapel Hill, Chapel Hill, North Carolina, United States of America; 3 Biobehavioral Health Department, The Pennsylvania State University, University Park, Pennsylvania, United States of America; 4 Population Health Sciences, Geisinger, Danville, Pennsylvania, United States of America; 5 Division of Human Genetics, Vanderbilt University Medical Center, Nashville, Tennessee, United States of America; 6 Department of Clinical Epidemiology, Leiden University Medical Center, Leiden, The Netherlands; 7 Novo Nordisk Foundation Center for Basic Metabolic Research, Faculty of Health and Medical Sciences, University of Copenhagen, Copenhagen, Hovedstaden, Denmark; 8 Charles Bronfman Institute for Personalized Medicine, Icahn School of Medicine at Mount Sinai, New York, New York, United States of America; 9 The Mindich Child Health and Development Institute, Icahn School of Medicine at Mount Sinai, New York, New York, United States of America; 10 Department of Anthropology, University of Toronto Mississauga, Mississauga, Ontario, Canada; 11 Unidad de Investigación Medica en Bioquímica Hospital de Especialidades, Centro Medico Siglo XXI, Mexico City, Mexico; 12 Department of Epidemiology, Emory University Rollins School of Public Health, Atlanta, Georgia, United States of America; 13 Atlanta VA Health Care System, Decatur, Georgia, United States of America; 14 Department of Medicine, Emory University School of Medicine, Atlanta, Georgia, United States of America; 15 Department of Biostatistics, Boston University School of Public Health, Boston, Massachusetts, United States of America; 16 Department of Epidemiology, University of Michigan School of Public Health, Ann Arbor, Michigan, United States of America; 17 Survey Research Center, University of Michigan Institute for Social Research, Ann Arbor, Michigan, United States of America; 18 Brown Foundation Institute of Molecular Medicine, McGovern Medical School, The University of Texas Health Science Center at Houston, Houston, Texas, United States of America; 19 Department of Epidemiology, Human Genetics and Environmental Sciences, The University of Texas Health Science Center at Houston School of Public Health, Houston, Texas, United States of America; 20 Preventive Medicine, University of Southern California Keck School of Medicine, Los Angeles, California, United States of America; 21 Cancer Prevention Institute of California, University of Southern California, Fremont, California, United States of America; 22 Division of Public Health Sciences, Fred Hutchinson Cancer Research Center, Seattle, Washington, United States of America; 23 Department of Statistics, Rutgers University, New Brunswick, New Jersey, United States of America; 24 The School of Public Health, University of Texas at Brownsville, Brownsville, Texas, United States of America; 25 Montréal Heart Institute, Montréal, Québec, Canada; 26 Department of Genome Sciences, University of Virginia, Charlottesville, Virginia, United States of America; 27 Division of Biomedical Informatics and Personalized Medicine, University of Colorado School of Medicine, Aurora, Colorado, United States of America; 28 Department of Epidemiology, Johns Hopkins University Bloomberg School of Public Health, Baltimore, Maryland, United States of America; 29 Massachusetts Veterans Epidemiology Research and Information Center, VA Boston Healthcare System, Boston, Massachusetts, United States of America; 30 Department of Medicine, Brigham and Women’s Hospital, Harvard Medical School, Boston, Massachusetts, United States of America; 31 Department of Human Genetics, University of Pittsburgh Graduate School of Public Health, Pittsburgh, Pennsylvania, United States of America; 32 Department of Medicine, Johns Hopkins Medicine, Baltimore, Maryland, United States of America; 33 Department of Human Medical Genetics and Genomics, University of Colorado Denver, Denver, Colorado, United States of America; 34 Department of Genetics, University of North Carolina at Chapel Hill, Chapel Hill, North Carolina, United States of America; 35 Department of Nutrition, University of North Carolina at Chapel Hill Gillings School of Global Public Health, Chapel Hill, North Carolina, United States of America; Newcastle University, UNITED KINGDOM OF GREAT BRITAIN AND NORTHERN IRELAND

## Abstract

Severe obesity (SevO) is a primary driver of cardiovascular diseases (CVD), cardiometabolic diseases (CMD) and several cancers, with a disproportionate impact on marginalized populations. SevO is an understudied global health disease, limiting knowledge about its mechanisms and impacts. In genome-wide association study (GWAS) meta-analyses of the tail end of the BMI distribution (≥95^th^ percentile BMI) and two SevO phenotypes [Obesity Class III BMI ≥ 40 kg/m^2^ and Obesity Class IV BMI ≥ 50 kg/m^2^] in 159,359 individuals across eleven ancestrally diverse population-based studies followed by replication in 480,897 individuals across six ancestrally diverse studies, we identified and replicated three novel signals in known loci of BMI [*TENM2*, *PLCL2*, *ZNF184*], associated with SevO traits. We confirmed a large overlap in the genetic architecture of continuous BMI and severe obesity phenotypes, suggesting little genetic heterogeneity in common variants, between obesity subgroups. Systematic analyses combining functional mapping, polygenic risk scores (PRS), phenome wide association studies (PheWAS) and environmental risk factors further reinforce shared downstream comorbidities associated with continuous measures of BMI and the importance of known lifestyle factors in interaction with genetic predisposition to SevO. Our study expands the number of SevO signals, demonstrates a strong overlap in the genetic architecture of SevO and BMI and reveals a remarkable impact of SevO on the clinical phenome, affording new opportunities for clinical prevention and mechanistic insights.

## Author summary

Severe obesity (SevO), which includes individuals with a body mass index (BMI) over 40 kg/m^2^, is a major cause of heart disease, diabetes, and some cancers. It affects marginalized communities at higher rates but remains under-researched. To better understand the biology behind severe obesity, researchers analyzed genetic data from over 159,000 people of diverse backgrounds and confirmed their findings in nearly 481,000 more individuals. The study identified three new genetic regions linked to severe obesity, within genes already known to influence body weight. Interestingly, the study showed that severe obesity and overall BMI share much of the same genetic background, meaning the genes that influence body weight also contribute to more extreme obesity. By combining genetic risk scores, health records, and environmental factors, the research highlighted how genetic predisposition and lifestyle together shape the risk for severe obesity and related health problems. This work expands our knowledge of the genetic factors behind severe obesity and emphasizes the importance of both genetics and the environment in tackling obesity-related diseases, offering new insights for prevention and treatment.

## Introduction

Severe obesity (SevO) (Body Mass Index [BMI] (≥40 kg/m^2^) is an emerging global health disease which imparts a substantial and growing morbidity and mortality burden that disproportionately affects underserved populations [1–3]. With marked heterogeneity in the disease of obesity (([BMI] ≥30 kg/m^2^)), severe obesity represents a higher degree of obesity than frank obesity, more extreme weight gain, and potential mechanistic differences [4]. In addition, the diagnostic definition of obesity is currently based on BMI thresholds with no information about health at the individual level [5] limiting inference on fat distribution and metabolic health [6–9]. Thus, obesity should be considered as a multifaceted disease that not only encompasses common covariates such as age, gender and ancestry but alternative measures of obesity and its manifestation to downstream disease [5]. However, SevO, is markedly different in terms of multi-organ dysfunction and has been directly correlated with a myriad of health complications including type 2 diabetes (T2D), coronary heart disease (CHD) and various forms of cancer [10,11]. Despite this, very few studies have explored the biological and genetic mechanisms underlying SevO. Indeed, most studies have grouped individuals with SevO with individuals classified according to World Health Organization (WHO) class I ([BMI] ≥30 kg/m^2^ to <35 kg/m^2^) or class II ([BMI] ≥35 kg/m^2^ to <40 kg/m^2^) obesity, or they have been excluded altogether in clinical and epidemiological studies [12], thereby masking possible detrimental effects of the critical SevO subtype. Thus, the health impacts for individuals at the highest end of the spectrum for obesity are underestimated or largely undocumented, limiting knowledge about the mechanistic pathways and impacts, including its genetic determinants. Genome wide association studies (GWAS) have mapped thousands of independent loci among people of diverse ancestries influencing both dichotomous ([BMI] ≥30 kg/m^2^ denotes individuals with obesity, [BMI] ≥18.5 kg/m^2 ^< 25 kg/m^2^ denotes normal weight individuals) and quantitative traits of BMI [13–17], offering insights into the genetic architecture of obesity. To date, the largest known population-based GWAS of SevO was performed in 263,407 individuals of European ancestry and identified seven novel loci associated with different classes of obesity; two of which (*HS6ST3* [heparan sulfate 6-O-sulfotransferase 3] and *ZZZ3* (zinc finger ZZ-type containing 3) were associated with WHO Obesity Class II ([BMI] ≥35 kg/m^2^) [18]. The remaining loci associated with BMI tails and clinical classes of SevO had variants that intersected with studies of continuous BMI GWAS [17,19] and mapped highly penetrant variants in loci that affect key molecular and neural pathways involved in human energy homeostasis [18,20–27]. Given that most SevO GWAS have been limited by small sample sizes [21,28], inconsistent phenotype definition (clinical classes of obesity, extremes of distribution tails) [29], a lack of participants of diverse backgrounds (with current analyses mainly including Europeans) [28,30], and a focus on monogenic causes of obesity that are difficult to detect in population-based samples [18], we sought to perform the largest known GWAS of SevO among individuals of diverse ancestries, leveraging extremes of obesity and WHO described clinical obesity classes. We hypothesized that given its extreme form, common variants for SevO could be mapped with smaller sample sizes than GWAS for other obesity related traits and that such variants would likely overlap with variants mapped using continuous BMI and other obesity binary traits. Lastly, we performed functional mapping, polygenic risk score analysis (PRS), phenome wide association studies (PheWAS), and assessed environmental risk factors in the context of predicted high risk for, to discern the biological mechanisms underlying body weight regulation.

## Results

### Demographic characteristics

Genotyping, imputation quality control, and statistical platforms used for analyses are detailed in S1 Table. We defined cases as individuals in the top 5th percentile of BMI, and controls in the 5th–50th percentile, stratified by sex after adjusting for age, age^2^, and ancestry (BMI cases ranged from 34 kg/m^2^ to 48 kg/m^2^ and BMI controls ranged from 22 kg/m^2^ to 27 kg/m^2^) (S1**–**S4 Figs). We also used the WHO classification to define SevO cases as individuals with BMI ≥ 40 kg/m^2^ (Obesity Class III) or ≥50 kg/m^2^ (Obesity Class IV), and controls as a BMI of 18.5–24.9 kg/m^2^. Baseline characteristics of our study stratified by ancestry and phase is presented in Table 1. While there are limitations of defining our controls within the 5^th^ to 50^th^ percentiles, we utilized a growth chart approach to mirror what is normally conducted by the Centers for Disease Control (CDC) and Prevention to define BMI categories in children [31]. In addition, we incorporated WHO guidelines of SevO (based on BMI thresholds) and showed consistent association of significant variants across all three traits (Table 2).

**Table 1:** Baseline characteristics of average BMI in cases and controls across all three phenotypic traits stratified by self-reported ancestry and stage.

Stage	Population Background	All		95th Percentile				Obesity Class 3				Obesity Class 4			
		N	Age (years)	CASE		CONTROL		CASE		CONTROL		CASE		CONTROL	
				N	BMI (kg/m^2)	N	BMI (kg/m^2)	N	BMI (kg/m^2)	N	BMI (kg/m^2)	N	BMI (kg/m^2)	N	BMI (kg/m^2)
**Discovery**	AA	23,237	51.34 (11.29)	1,860	50.72 (6.98)	16,503	26.61 (2.67)	4,813	46.74 (6.63)	6,685	22.75 (1.62)	1,051	56.86 (6.94)	6,041	22.72 (1.64)
	AI	276	58.97 (7.34)	26	45.99 (5.66)	231	25.73 (2.21)	45	44.14 (4.93)	124	22.92 (1.54)	0	N/A	0	N/A
	AS	4,138	55.86 (10.06)	371	35.69 (3.74)	3,386	22.60 (1.54)	33	42.81 (2.27)	1,973	22.46 (1.63)	0	N/A	0	N/A
	EA	108,843	50.96 (12.73)	8,076	51.90 (6.99)	71,607	27.10 (2.60)	25,037	47.07 (6.79)	28,324	22.67 (1.60)	6,072	56.67 (6.72)	26,021	22.79 (1.58)
	HA	20,079	51.22 (11.48)	1,740	45.25 (5.30)	15,803	25.70 (2.05)	2,170	45.38 (5.39)	7,285	22.85 (1.54)	306	55.91 (5.55)	5,405	22.82 (1.59)
	HW	2,232	54.00 (7.35)	191	45.00 (5.96)	1,710	25.18 (1.94)	231	44.88 (4.83)	1,015	22.82 (1.50	28	54.74 (3.87)	924	22.82 (1.49)
	MA	508	44.38 (14.42)	47	42.19 (6.03)	451	23.25 (1.91)	29	45.16 (5.92)	394	22.34 (1.66)	0	N/A	0	N/A
	**Subtotal**	**159,313**		**12,311**		**109,691**		**32,358**		**45,800**		**7,457**		**38,391**	
**Replication**	AA	62,539	54.87 (10.28)	5,987	45.87 (4.64)	53,908	26.32 (2.28)	8,534	44.61 (4.46)	20,516	22.73 (1.58)	939	54.31 (4.34)	20,340	22.73 (1.58)
	EA	390,569	57.21 (9.61)	38,532	42.34 (4.14)	347,075	25.08 (1.78)	29,453	44.31 (4.25)	201,828	22.85 (1.53)	2,811	54.3 (4.24)	201,077	22.85 (1.53)
	EAS	1,249	52.23 (7.80)	125	33.45 (2.55)	1,124	22.14 (1.31)	0	N/A	0	22.28 (1.68)	0	N/A	0	N/A
	HA	21,781	50.89 (13.88)	2,108	45.40 (4.51)	19,037	26.98 (1.99)	2,744	44.42 (4.35)	5,314	23.02 (1.47)	275	54.55 (4.22)	5,314	23.02 (1.47)
	SAS	4,759	53.52 (8.46)	473	38.96 (3.91)	4,286	24.37 (1.45)	129	43.87 (4.21)	3,080	22.96 (1.52)	0	N/A	0	N/A
	**Subtotal**	**480,897**		**47,225**		**425,430**		**40,860**		**230,738**		**4,025**		**226,731**	
**Grand Total**		**640,210**		**59,536**		**535,121**		**73,218**		**276,538**		**11,482**		**265,122**	

**Table 2:** Summary of unreported independent GWAS signals of severe obesity traits in the discovery and replication cohorts. Independent signals were defined as lead variants having met a genome-wide significant *P* value (*P* < 5 × 10^−8^) in our discovery cohort and a Bonferroni adjusted significance (*P = *0.005) in the replication cohort across any strata (all, men, or women) had a linkage disequilibrium (LD) *r*^2^ < 0.6 with other variants and were defined as those independent from each other at *r* ^*2* ^< 0.1 inside a subset of independent significant variants. Genes were annotated based on ±250kb of the lead variant and/ or closest gene present.

Marker (Chr:Position hg19: Effect Allele: Other Allele)	Genes (±250kb of the lead variant)	rsid	Strata 95% tile	*Discovery OR (95%CI)	^¶^Replication OR (95%CI)	Strata ObClass3	*Discovery OR (95%CI)	^¶^Replication OR (95%CI)	Strata ObClass4	*Discovery OR (95%CI)	^¶^Replication OR (95%CI)
**3: 5944942:T:C**	*BHLHE40-AS1*	rs17824177	All	0.91 (0.88, 0.94)	0.96 (0.94,0.98)	All	0.89 (0.86, 0.92)	0.96 (0.94,0.98)	All	0.89 (0.84, 0.94)	0.96 (0.9,1.02)
			Men	0.92 (0.87, 0.98)	0.96 (0.94,0.98)	Men	0.86 (0.81, 0.92)	0.95 (0.92,0.97)	Men	0.90 (0.81, 1.00)	0.94 (0.87,1.01)
			Women	0.91 (0.87, 0.95)	0.96 (0.93,0.99)	Women	0.91 (0.87, 0.94)	0.98 (0.94,1.02)	Women	0.89 (0.83, 0.95)	1.03 (0.91,1.15)
**3: 17115122:C:G**	*PLCL2*	rs36118680	All	1.05 (1.01, 1.10)	1.03 (1.01,1.05)	All	1.10 (1.06, 1.14)	1.03 (1.01,1.06)	All	1.09 (1.02, 1.16)	1.05 (0.99,1.12)
			Men	1.02 (0.96, 1.09)	1.02 (1.00,1.05)	Men	1.03 (0.97, 1.11)	1.04 (1.01,1.06)	Men	0.96 (0.86, 1.08)	1.06 (0.98,1.13)
			Women	1.07 (1.02, 1.13)	1.05 (1.02,1.08)	Women	1.13 (1.08, 1.18)	1.03 (0.99,1.07)	Women	1.14 (1.06, 1.22)	1.04 (0.91,1.17)
**5: 166756351:T:C**	*TENM2*	rs13155681	All	1.10 (1.07, 1.14)	1.04 (1.02,1.05)	All	1.06 (1.03, 1.09)	1.04 (1.02,1.06)	All	1.11 (1.06, 1.16)	1.04 (0.99,1.09)
			Men	1.13 (1.08, 1.18)	1.03 (1.01,1.04)	Men	1.08 (1.03, 1.14)	1.04 (1.02,1.06)	Men	1.14 (1.05, 1.25)	1.05 (1.00,1.11)
			Women	1.09 (1.05, 1.13)	1.06 (1.03,1.08)	Women	1.05 (1.01, 1.08)	1.03 (1.00,1.06)	Women	1.09 (1.04, 1.15)	1.01 (0.91,1.11)
**6: 27449233:A:G**	*ZNF184,* *POM121L2* *PRSS16* *ZNF391*	rs140919115	All	1.29 (1.09, 1.51)	1.24 (1.15,1.33)	All	1.36 (1.18, 1.58)	1.18 (1.10,1.27)	All	1.33 (1.02, 1.74)	1.29 (1.06,1.51)
			Men	0.77 (0.56, 1.06)	1.22 (1.12,1.32)	Men	0.79 (0.56, 1.11)	1.18 (1.08,1.28)	Men	0.88 (0.45, 1.74)	1.11 (0.84,1.38)
			Women	1.61 (1.33, 1.96)	1.29 (1.11,1.46)	Women	1.66 (1.40, 1.96)	1.23 (1.05,1.41)	Women	1.45 (1.07, 2.01)	1.98 (1.58,2.37)

*Discovery Significance is determined by GWAS significance threshold (5x10^-8^)

We identified independent index SNPs based on two thresholds. We first defined index SNPs meeting genome-wide significance (P ≤ 5 × 10 ⁻ ⁸) within ±250kb (used to determine the borders of genomic risk loci) in linkage disequilibrium (LD: r^2^ < 0.6) using the 1000 Genomes Project reference. If multiple independent SNPs reached significance within the predefined locus (using an r^2^ < 0.1 for the second clumping), each SNP was functionally annotated. However, it should be noted that it is unclear whether SNPs within the ± 250kb window of the independent SNP and an r^2^ of 0.6 are considered separate when using an r^2^ threshold of 0.1. Therefore, a complementary conditional analysis was conducted using known BMI SNPS from different ancestry panels (EUR, AFR, HIS and EAS) to assess the independence of SNPS initially considered the index SNP.

**Fig 1:**
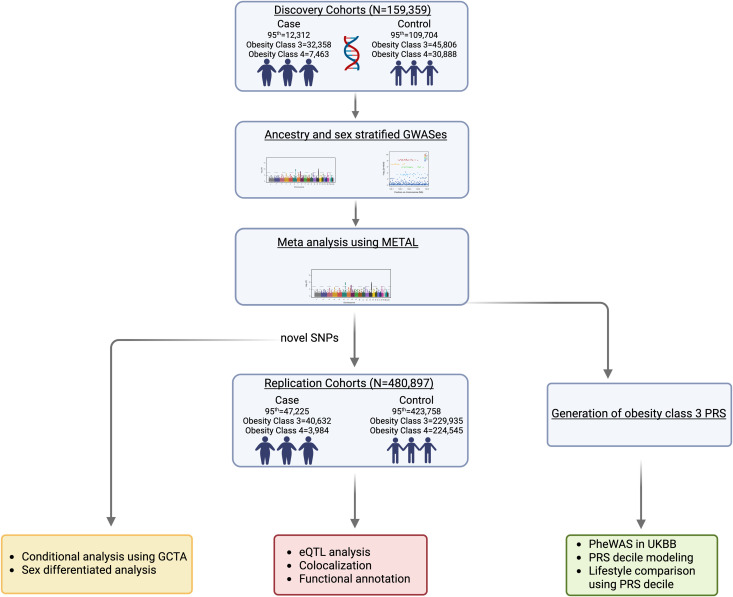
Schematic of the study design for genomic analysis and systematic comparisons of SevO in ancestrally diverse populations (Fig 1 created in biorender (2025, https://BioRender.com/f9g0w8p, ZT28HSBHBH.).

We also considered rare variants (MAF < 1%) given their potential higher frequencies in ancestry-stratified analyses, though acknowledging the unreliability of rare variants genotyped by SNP arrays including dependency on reference genome across multi-ancestry populations [32] and ascertainment bias [33]. Stage 1 discovery and Stage 2 replication analyses included 11 and 6 population-based studies, with a total of 159,359 and 480,997 individuals across diverse ancestries respectively (Fig 1 **and** Tables 1 **and** S2–S4).

### GWAS of severe obesity (SevO) traits

We analyzed >31 million genotyped or imputed SNPs in ancestry-, sex-specific, and combined analyses (**Figs 5 and** S5–S13). Initially, in the combined ancestry and sex analysis, we identified four novel signals not located near known loci of obesity (*BHLHE40-AS1*, *PLAS2R1*, *PIWL1* and *CDHR1*) and six novel signals in or near known obesity loci (*PDGFC, SMARCAD1, TENM2, ZNF184, PLCL2,* and *SLC39A11)* at genome-wide significance (P < 5 × 10 ⁻ ⁸) (S5 Table). SNP-based heritability in our discovery phase combined ancestry samples was 0.27 (*P* = 2.39x10^-97^) and 0.29 (*P* = 5.37x10^-50^) for WHO Class III and Class IV obesity respectively (S32 Table). Both SevO classes also showed high genetic correlation with BMI (rg = 0.94 [*P* = 2.22x10^-1096^] for Class III, rg = 0.94 [*P* = 4.60x10^-443^] for Class IV).

**Fig 2:**
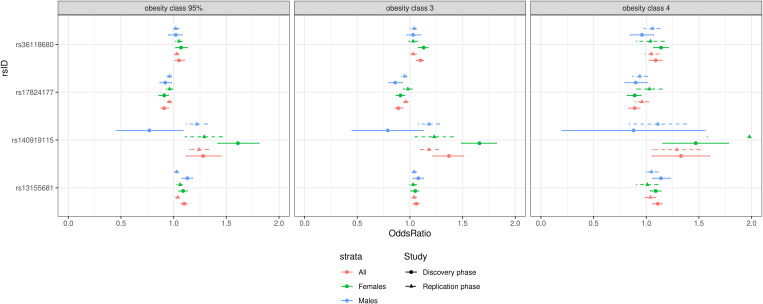
Forest-plot illustrating the direction of association for the four validated variants (discovery and replication) across the three obesity classes (95% class, Obesity Class 3 and Obesity Class 4) for each stratum (all, females and males).

**Fig 3:**
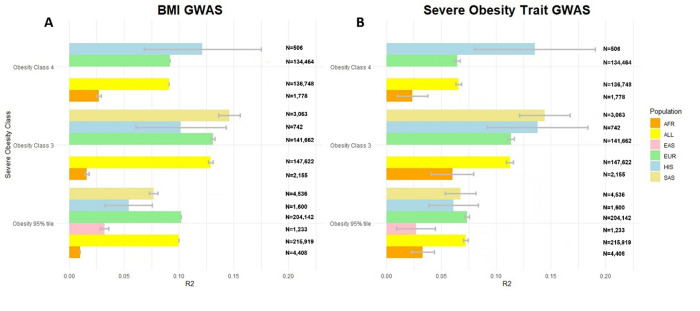
Phenotypic variance explained (R2) by (A) using HapMap 3 SNPs in the BMI GWAS from the GIANT consortium and (B) using the PRS-CS method with HapMap3 consortium SNPs for each of the SevO GWAS traits (e.g., 95% Obesity, Obesity Class III and Obesity Class IV). Using approximately 1/3 the sample size in our SevO PRS we show similar predictive power in explaining phenotypic variance of SevO traits when compared to the PRS generated from the BMI GWAS.

**Fig 4:**
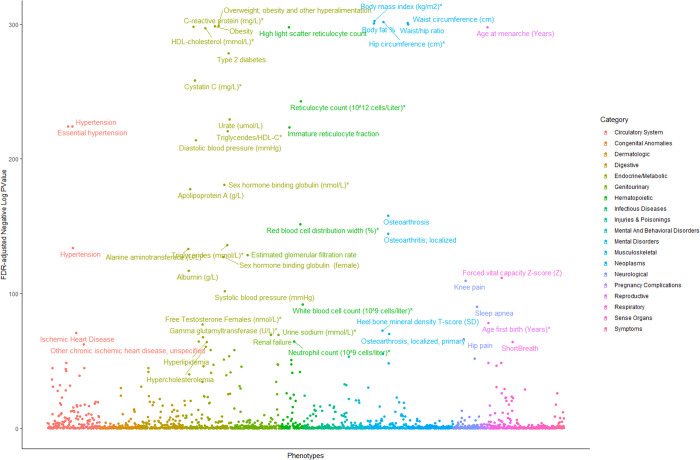
PheWAS analysis of secondary phenotypes (max 1668) within the UKBB Europeans with our Obesity Class III PRS derived from our discovery analyses.

We then performed secondary stepwise conditional analyses correcting for effect and *P* values of variants (across a sliding window of 10Mb) with known BMI variants based on LD from four ancestries (EUR, AFR, HIS and EAS) (S6 Table). A secondary signal is affirmed if any new variants meet our Bonferroni adjusted *P*-value of 5x10^-8^. Of the 10 variants 8 variants (in or near *PLA2R1*, *PLCL2*, *PDGFC*, *TENM2, ZNF184, CDHR1, PIWIL1* and *SLC39A11*) remained independent after conditioning across all four reference panels. Of interest is the variant rs17824177 in *BHLHE40-AS1*, for which we initially deemed a novel locus finding. However, with recent new data available we showed that rs17824177 was in partial LD with known BMI variants in *BHLHE40-AS1* within the European refence panel (S6 Table). Despite this, we decided to keep this as a novel discovery and provide additional downstream analysis for *BHLHE40-AS1* highlighting its importance in the context of severe obesity (see **Expression Quantitative Trait Loci (eQTL), colocalization** section).

Lastly, we performed sex-differentiated analysis across our discovery phase. We showed no differences between sex (using a genome-wide *P* value threshold) with our 10 novel variants. However, two variants in or near known loci of BMI (rs146595723 in *PTPN3* and rs1966846 near *SYT12*) exhibited genome-wide differences between sex (S7 Table). Interestingly, the effect sizes were larger across our male participants (rs146595723_*PTNP* [β = 0.395, *P* = 0.002, *P*_*sex*_ = 5.65x10^-4^] and rs1966846_*SYT12* [β = -0.340, *P* = 0.004, *P*_*sex*_ = 9.49x10^-3^] despite males having lower statistical power in our discovery cohort.

These ten variants were carried forward to Stage 2 replication. We replicated the *BHLHE40-AS1* signal and three novel signals near known loci (*TENM2, ZNF184, PLCL2*) in a meta-analysis using a Bonferroni-adjusted threshold of *P* < 0.005 (Table 2 **and** Fig 2). All variants showed consistent effect directions across SevO classes. Ancestry-stratified summary statistics for these 10 variants are in S8–S13 Tables.

Note initial GWAS using the discovery cohort identified 10 novel variants associated with severe obesity classes. Here we show the 4 variants that were identified in our discovery cohort and were validated in our replication cohort using Bonferroni adjusted threshold of 0.005. For full results for all 10 variants for both discovery and replication cohort please S5 Table.

### Expression Quantitative Trait Loci (eQTL), colocalization and functional annotations of novel GWAS findings

We evaluated the functional and clinical significance of our four variants identified in Stage 1 discovery and validated in Stage 2 replication using colocalization with expression quantitative trait loci (eQTLs) from GTEx [34], eQTLGen Phase 1 [35], and a large Mexican American whole blood eQTL resource. No eQTLs were found in eQTLGen Phase 1. In GTEx, the minor (C) allele of rs36118680 was associated with increased *PLCL2* expression in whole blood (NES = 0.28, *P* = 6.3 × 10 ⁻ ¹⁵) (S14 Table). In the Mexican American dataset, the minor (C) allele of rs17824177 was associated with higher *BHLHE40-AS1* expression (NES = 0.395, P = 0.01) (S15 Table**).** Colocalization modeling with Obesity Class III GWAS and GTEx expression data revealed colocalization between rs36118680 and *PLCL2* expression in whole blood (posterior probability = 99%). However, there was no evidence of colocalization between rs17824177 and *BHLHE40-AS1* expression (posterior probability = 33%) from our Mexican American whole blood eQTL resource. The remaining two variants (rs13155681 and rs140919115) were absent from the publicly available expression databases. We also examined the potential functional significance of our four variants that reached significance level using the combined annotation-dependent depletion (CADD) method. We showed that rs17824177 (*BHLHE40AS1*) and rs140919115 (*ZNF184*) had a CADD-scaled C score of more than 10 suggesting these variants were considered deleterious (~5%) and should be prioritized in downstream analysis (S17 Table).

### Plausible biological roles for genes surrounding novel replicated variants

Many of the genes surrounding these index variants (±250kb of the index variant or closest gene present) had important roles in the pathogenesis of SevO including neuronic control of food intake, sustained low-grade chronic inflammation and adipogenesis. A summary of the plausible biological roles of genes surrounding the four novel variants with SevO using online resources and a comprehensive manual review of the published literature is presented in S16 Table.

### Overlap with known BMI loci

We assessed the relevance of known BMI loci for Obesity Class III by examining the concordance of direction among variants identified in a BMI meta-analysis of the UK Biobank and GIANT and the top 714 BMI variants from COJO analysis with our ancestry combined Obesity class III discovery results [17]. We explored concordance using three different *P* value thresholds (0.05, 0.005 and 0.0005) to investigate the broader genetic architecture. We showed extremely significant concordance rates between the UK Biobank and GIANT BMI meta-analysis with our SevO GWAS results across all three *P* value thresholds in both the meta-analysis (S18 Table) and top COJO 714 BMI signals (S19 Table) indicating a strong enrichment of susceptibility alleles across traits.

We also analyzed our four validated novel variants (or their LD proxies, R^2^ > 0.8) in the GIANT+UKBB [17] In GIANT+UKBB by Yengo *et al*, only rs17824177 showed some evidence of association with BMI (*P* = 2.6 × 10 ⁻ ⁶), though not genome-wide significant. The other variants were absent due to their rarity or the older HapMap imputation panel.

### PRS-estimation of SevO

PRS were calculated using two methods: PRS-CS with HapMap3 SNPs for SevO traits (e.g., 95^th^ percentile BMI, Obesity Class III, and Class IV) and BMI GWAS results from the GIANT consortium (N ~ 300k^19^), and genome-wide significant independent variants from SevO GWAS traits in this study. We tested the PRS on phenotypic and genotype data from 235,071 unrelated UKBB participants and 3,063 unrelated Mexican Americans from the CCHC study, all classified as cases or controls for SevO. The participants included 223,699 Europeans, 4,057 African or African Americans, 1,537 East Asians, 4,978 South Asians, and 3,063 Mexican Americans. PRS performance by population is shown in Fig 3 and S20 Table.

As expected, R^2^ estimates were low when using only GWAS-significant SNPs to calculate PRS (S20 Table). However, the PRS-CS method with HapMap 3 variants improved R^2^ across all ancestries. For Obesity Class III, R^2^ improved to 6.0% for African participants, 11.4% for Europeans, 13.8% for Hispanics, and 14.4% for South Asians. For the 95th percentile BMI, R^2^ was 3.3% for Africans, 2.7% for East Asians, 7.4% for Europeans, 6.1% for Hispanics, and 6.8% for South Asians. For Obesity Class IV, R^2^ was 2.4% for Africans, 6.5% for Europeans, and 13.5% for Hispanics (S20 Table). Overall, PRS performance was highest for Obesity Class III, except in the Hispanic population. We also calculated PRS using BMI GWAS results and compared the performance to SevO GWAS-based PRS. R^2^ performance was similar across ancestries, though the SevO GWAS had a smaller sample size (~120,000 for the 95th percentile, ~ 80,000 for Obesity Class III, ~ 50,000 for Class IV) compared to the BMI GWAS (~300,000) (S20 Table). Only three East Asian participants had BMI ≥ 40 kg/m^2^, and no East or South Asian participants had BMI ≥ 50 kg/m^2^, so PRS were not calculated for these groups.

We assessed the correlation between our all ancestry SevO Class III PRS with the BMI PRS and found moderate evidence of correlation (R = 0.57). When stratifying by ancestry in unrelated individuals we showed varying levels of correlation ranging from 0.13 to 0.63 (S21 Table). We also checked the proportion overlap between individuals in our low susceptibility (SevO vs. BMI) and high susceptibility (SevO vs. BMI) PRS’s and found approximately 1/3 of individuals overlap in the low and high 10^th^ percentiles for both PRS’s. This suggests that the SevO and BMI PRS’s are not capturing the same phenotype information reinforcing that SevO should be considered a separate phenotype from traditional obesity (BMI > 30 kg/m^2^).

### PRS-PheWAS in the UKBB

We conducted a phenome-wide association study (PheWAS) of PRS-SevO across diverse populations in the UKBB (S14 Fig **and** S24–S28 Tables). Obesity Class III PRS was associated with 37% of phenotypes (out of 1,668) in Europeans, including known obesity-related traits like BMI, body fat, waist circumference, metabolic comorbidities, and bone density. We also found strong associations with inflammation markers (C-reactive protein, gamma-glutamyltransferase) and hematopoietic traits (immature reticulocyte fraction, reticulocyte count) (Fig 4 **and** S24–S28 Tables).

### PRS deciles and lifestyle behavior modelling

A recent PRS for BMI, based on 2.1 million genetic variants, identified individuals at risk of SevO [36]. Khera et al. found that their BMI PRS explained 8.4% of BMI variation, with those in the high susceptibility decile (90^th^ percentile of the BMI PRS) had a higher average BMI of 2.9 kg/m^2^ and a 4.2 fold increased risk of SevO than those in the lower susceptibility decile (10^th^ percentile of the BMI PRS). We replicated this approach with our SevO PRS-CS risk score, separating into deciles (1–10) according to obesity categories using BMI thresholds: (1) BMI < 25 kg/m^2^ (normal weight), (2) BMI ≥ 25 kg/m^2^ to BMI < 30 kg/m^2^ (overweight), (3) BMI ≥ 30 kg/m^2^ to BMI < 40 kg/m^2^ (obesity) and (4) BMI ≥ 40 kg/m^2^ (SevO) (S29 Table).

**Fig 5:**
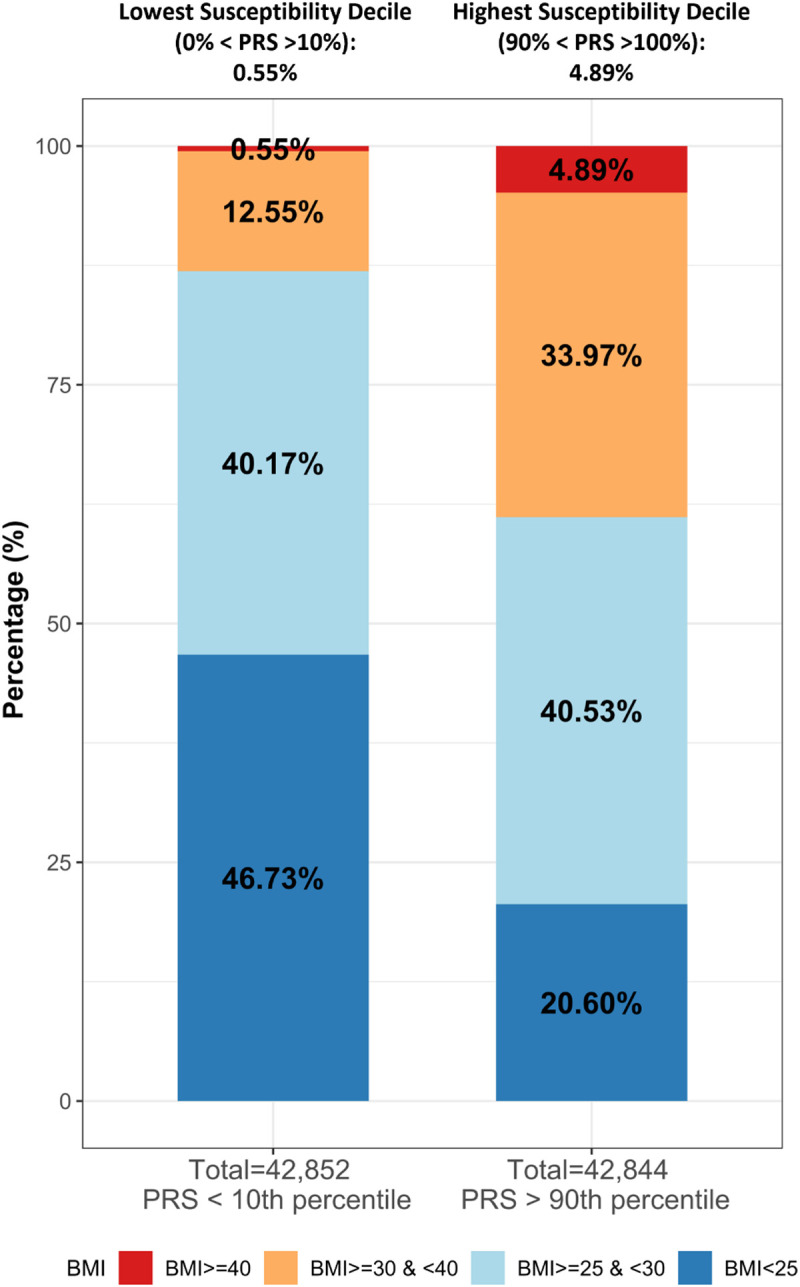
Relationship of SevO PRS > 90^th^ percentile with < 10^th^ percentile across BMI categories in ancestry -combined samples from the UKBB.

We then assessed the predictive power of our SevO PRS (90^th^ percentile vs. 10^th^ percentile) on Obesity Class III, in our ancestry -specific and -combined samples from the UKBB (S30 Table **and** S15–S16 Figs). Focusing on the combined samples, we found that SevO was present in 4.9% of those >90^th^ percentile for PRS compared to 0.55% of those in <10^th^ percentile category, corresponding to a 10-fold increased risk of SevO (Fig 5). These differences in distribution indicate a different pattern of BMI distribution across the tail ends of the PRS distribution. S33 Table provides estimates of the specificity, sensitivity, and positive and negative predictive values of our SevO PRS for Obesity Class III across the European, African, and South Asian samples from UKBB and the Hispanic samples from the CCHC cohort. The predictive power of the SevO PRS, assessed using receiver operating characteristic (ROC) curves, showed AUC values ranging from 0.62 to 0.77 when using PRS alone, and improved to 0.75 to 0.83 when combined with covariates across all ancestries. Sensitivity for Obesity Class III ranged from 15% to 39%, with positive predictive values from 16% to 33%.

**Fig 6:**
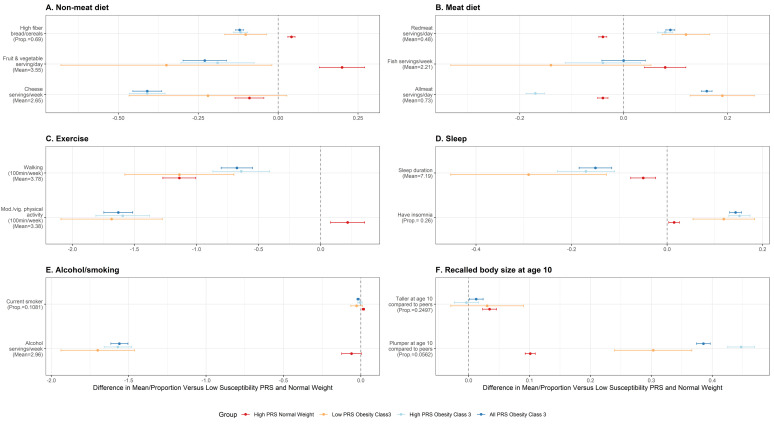
Lifestyle modeling comparisons between sample groups defined by PRS upper and lower deciles and BMI categories. We compared individuals having a low susceptibility PRS (<10^th^ percentile) and a BMI < 25 kg/m^2^ with 1) “Resilient” group [PRS in the ≥ 90^th^ percentile and a BMI < 25 kg/m^2^], 2) < 10^th^ percentile for PRS and SevO, 3) >90^th^ percentile for PRS and SevO Class III and 4) all PRS and SevO Clas III with different lifestyle factors including dietary, physical activity and sleep patterns, alcohol and smoke servings and perceived body size at age 10.

We also compared the pattern of association of individual lifestyle factors with BMI, within each of the PRS groups (Fig 6 **and** S31 Table). Of particular interest were participants with a PRS in the ≥ 90^th^ percentile and a BMI < 25 kg/m^2^, which we labeled the “Resilient group”. We compared individuals having low susceptibility PRS (<10^th^ percentile) and are of normal weight (BMI < 25 kg/m^2^) with 1) “Resilient” group [PRS in the ≥ 90^th^ percentile and a BMI < 25 kg/m^2^], 2) < 10^th^ percentile for PRS and SevO, 3) >90^th^ percentile for PRS and SevO Class III and 4) all PRS and SevO Clas III. The “Resilient group” reported healthier lifestyle behaviors when compared to participants with SevO or even those with BMI < 25 kg/m^2^ and with PRS < 10^th^ percentile, including improved dietary patterns (lower red meat intake, increased fruit, vegetable, fiber and fish intake), and increased moderate to vigorous physical activity (Fig 6 **and** S31 Table). Paradoxically, the “Resilient group” had lower mean sleep duration and a higher proportion of insomnia, despite much research showing the health benefits of improved sleep with metabolic outcomes. The “Resilient group” also smoked slightly more and consumed more alcohol than participants with SevO but consumed less alcohol than those with BMI < 25 kg/m^2^ and a PRS in the < 10^th^ percentile range (Fig 6 **and** S31 Table). The “Resilient group” was also less likely than participants with SevO but more likely than those with BMI < 25 kg/m^2^ and a PRS in the < 10^th^ percentile range to say they were ‘plumper’ than their peers at age 10 years (Fig 6 **and** S31 Table).

## Discussion

In our meta-analysis of GWAS of up to 159,359 individuals across 11 ancestrally diverse population-based studies, we identified numerous loci associated with SevO, many of which overlap with Class I obesity (BMI ≥ 30 kg/m^2^). Ten unreported independent variants in novel and known loci were associated with obesity related traits for the first time; four (rs17842177, rs36118680, rs13155681 and rs140919115) of these were validated in our replication cohort of 480,897 individuals of diverse ancestries. Conditional analysis showed 8 of our 10 variants remained independent. Of interest is the variant rs17842177 in *BHLHE40-AS1*, for which we initially considered it a novel locus of severe obesity but recently discovered the variant to be in partial LD with known BMI variants in Europeans. Despite this we still considered it potentially novel and provided additional downstream analysis highlighting its importance in the context of severe obesity. We lastly conducted sex-differentiated analysis and found two variants in known loci of BMI exhibiting genome wide sex differences reinforcing the importance of understanding of sexual dimorphism across complex traits. Our study expands the number of identified SevO signals, confirms strong overlap in the genetic architecture of SevO and BMI and reveals a remarkable impact of SevO on the clinical phenome, affording new opportunities for mechanistic insights and clinical prevention and intervention. These findings further enhance the underlying biology of SevO and highlight novel genetic signals not previously implicated in SevO pathophysiology.

Before severe obesity became as common as it is today, the focus was on monogenic forms of the disease. It is only more recently that the form of common complex severe obesity became more prevalent in the population. Thus, early genetic studies of severe obesity (SevO) focused on identifying gene-disrupting mutations in the leptin-melanocortin pathway (e.g., *LEPR, MC4R, POMC*) [37–41], which are linked to early-onset SevO through dysregulation of food intake and food preferences [42]. While these studies provided novel mechanistic insights, GWAS have shifted focus to polygenic forms of obesity. To date, GWAS has identified over 1,000 loci influencing BMI, with some high-impact variants near genes associated with extreme and early-onset obesity [16,19,43–46] also enriched in polygenic obesity traits.

GWAS of SevO found numerous susceptibility loci that intersected with earlier-identified BMI loci including the archetypal *FTO, MC4R*, *SH2B1* and *NPC1* suggesting little etiological heterogeneity between obesity subgroups [21,22,24]. However, many of these GWAS had small sample-sizes, low coverage of markers tested, and inconsistent phenotype groupings, all which may have limited study findings. A large GWAS involving 263,407 individuals of European ancestry identified 165 genetic loci associated with BMI, height, waist-to-hip ratio, and WHO-defined obesity classes: Overweight (BMI ≥ 25 kg/m^2^), Obesity Class I (BMI ≥ 30 kg/m^2^), Obesity Class II (BMI ≥ 35 kg/m^2^), and Obesity Class III (BMI ≥ 40 kg/m^2^) [18]. Of these, only one known BMI variant, rs1421085 in the *FTO* gene, was significantly associated with Obesity Class III in the discovery phase [OR=1.45, *P = *3.93 X 10^-26^] and was validated in the replication cohort [OR=1.47, *P = *2.11 X 10^-14^] in this study. This variant was also replicated in our GWAS of SevO and in other smaller genetic studies of SevO. Additionally, 22 variants were associated with Obesity Class II, including two novel loci in *HS6ST3* (heparan sulfate 6-O-sulfotransferase 3) and ZZZ3 (zinc finger ZZ-type containing 3). The absence of other known or novel associations with Obesity Class III in this study likely reflects low statistical power, with 3,986 cases and 67,010 controls. In our discovery GWAS, we found validation for the novel Obesity Class III traits reported by Berndt *et al.* for *HS6ST3* (rs7989336) and *ZZZ3* (rs17381664), particularly in Obesity Class IV, as well as in ancestry-combined and female-specific samples.

Consistent with findings in other polygenic traits, our study shows that large sample sizes are needed to identify variants associated with population extremes [18,47]. Using a population-based design, we identified a novel locus (*BHLHE40-AS1*) and three unreported independent signals in known BMI loci linked to SevO traits. These loci are involved in inflammation, food intake regulation, and altered adipose differentiation. Despite the strong genetic overlap between BMI and SevO (r_g_s > 0.80), focusing on trait extremes allowed us to detect novel signals with smaller sample sizes.

Obesity is often characterized by low-grade inflammation that is associated with a sequalae of metabolic diseases including type 2 diabetes, hypertension, and cardiovascular complications [48]. As an acute-phase reactant to inflammation and infection, C-reactive protein (CRP) is associated with obesity [49] along with inflammatory mediators such as *TNF-α* and *IL-*6 [50]. Our study identified the novel *BHLHE40-AS1* as a strong candidate gene of SevO both through our GWAS findings and replication but also through eQTL evidence in whole blood samples from Mexican American CCHC participants [51]. We examined the potential functional significance of a SNP using the CADD score to measure deleteriousness of a given variant which could lead to prioritizing causal variants. In this study we report a CADD score > 10 for rs17824177 (C_score_ = 15.27) which is predicted to be in the top ~ 5% most deleterious substitutions carrying a moderate level of functional importance. Recently, a study has demonstrated that *BHLHE40-AS1* modulates pro-inflammatory cytokines and is an important mediator of IL6/STAT3 signaling [52]. In addition, common variants in *BHLHE40-AS1* associate with gamma glutamyl transferase, a biomarker positively correlated with C-reactive protein [53] and increased oxidative stress [54], which is supported by our PheWAS of Obesity Class III trait in UKBB. Our study suggests that *BHLHE40-AS1* is a strong biological candidate of obesity.

Accumulating evidence also suggests that distinct psychiatric disorders including schizophrenia and major depressive disorders share a common genetic etiology with obesity [55]. The intronic variant, rs140919115 (n.265 + 6212G > A), located in *ZNF184* was associated with Obesity Class III. This variant surrounds a locus that harbor genes (*ZNF391*, *POM121L2* and *PRSS16I*) involved with a plethora of mood-related disorders (S16 Table) [56,57]. This suggests a multi-faceted association between psychiatric disorders and BMI, involving pathways that may influence ‘binge’ eating and canonical food-related behaviors. In addition, *ZNF184* was found to amplify *FTO* gene expression levels [58], and may regulate body through *FTO*-mediated browning and mitochondrial thermogenesis [59].

Two additional loci (S16 Table) highlight genetic mechanisms involved in the developmental shift from brite (brown) adipocytes to energy-storing white adipocytes leading to reduced thermogenesis and increased lipid deposition [42]. The intronic variant, rs13155681 (c.226 + 44283C > T) located in *TENM2* was associated with the 95^th^ percentile BMI and may mediate obesity through adipocyte differentiation. *TENM2*, involved in regulating synaptic plasticity of neurons, is known to maintain white adipocyte phenotype, and reduced mitochondrial respiration during adipogenic differentiation [60] leading to increased storage of fat. The intronic variant rs36118680 (c.3204 + 5565C > G) was associated with Obesity Class III. The *PLCL2* locus mediates several biological functions, particularly those related to protein phosphatases. and is known to modulate obesity through fat lipolysis and thermogenesis regulation of adipocytes [61,62]. However, to translate genomic findings to meaningful outcomes, incorporation of other ‘omic’ platforms combined with advanced computational tools are needed to provide more proximal insight into the dysregulation of SevO biology in response to genetic modifications.

The SevO PRS in UKBB explained 11.4%, 6.0%, 13.8% and 14.4% of the phenotypic variance for Europeans, Africans, Hispanics and South Asian participants, respectively and was similar with the PRS generated using HapMap 3 SNPs in the BMI GWAS from the GIANT consortium [19]. Notably, we had similar predictive power and performance in BMI risk assessment with just 1/3 the sample size across all ancestries. In applying our SevO PRS in PheWAS, 37% of phenotypes demonstrate significant associations (max 1668). These findings highlight a broader impact of SevO on disease morbidity and mortality than previously anticipated. Notable associations were also found with hematopoietic phenotypes, such as immature reticulocyte fraction and reticulocyte count. Genes upregulated in obesity are selectively expressed in reticulocytes [63], aligning with studies showing higher red blood cell counts in obese individuals [64,65]. We also compared and found consistent associations between our results along with Robinson *et al* (2022) study that combined genomic and phenomic approaches in determining the genetic burden of obesity across other disease classes [66]. They highlighted that SevO Class III was associated with approximately 60% (433 additional phenotypes) of all severe obesity billing codes and their BMI PRS accounted for 7.5% variance in BMI and was associated with 296 clinical diseases [66]; many of which overlapped with our PheWAS of SevO Class III PRS in the UKBB. Consistent associations between traditional BMI (>30 kg/m^2^) PRS with our SevO PRS reinforces the important role of genetic predisposition plays across a spectrum of diseases and the overall healthcare burden imposed by obesity. Future research will explore pleiotropic genetic effects to better understand the link between obesity and its comorbidities.

A recent study demonstrated that PRS can identify individuals at high risk of obesity. By analyzing over 2 million variants in 300,000 people, the PRS explained 8.4% of BMI variation [36], with those in the top decile having a BMI 2.9 kg/m^2^ higher on average, and a 4.2-fold increased risk of SevO compared to the lowest nine deciles. In our SevO PRS for which we had a smaller subset of variants and a reduced sample size, we found a different BMI distribution at the tail ends of genetic susceptibility, highlighting the potential for heterogeneity in the relationship between PRS and BMI distribution. We found SevO in 4.9% of individuals in the top 90th percentile vs. 0.55% in the bottom 10th percentile, indicating a 10-fold increased risk. However, our study and previous studies showed low predictive value, with PPV values ranging from 0.16 to 0.33 and sensitivity from 0.15 to 0.39 [36,44]. This poor prediction may stem from the exclusion of lifestyle factors and selective participation bias in the UKBB. Our analysis suggests that incorporating lifestyle factors, like diet and physical activity, is crucial for improving personalized risk prediction.

There were several limitations to our study. Sample sizes were small for certain populations (e.g., East Asians) and SevO classes (Obesity Class IV). There was also a sex imbalance between the predominantly female discovery cohort and predominantly male replication cohort, limiting validation of sex-specific associations. Additionally, the 95% percentile BMI trait showed heterogeneity, lower predictability, and less heritability, which may have reduced GWAS power. Despite this, we conducted a complementary GWAS using WHO SevO classification to identify additional variants. Lastly, we recognize that stratification based on self-reported background may not fully align with genetic similarity, but it was necessary due to limitations in existing cohorts.

Despite these limitations, our study identified a novel locus influencing SevO and expands the number of identified SevO signals. We also confirmed a strong overlap between the genetic architecture of SevO and BMI. The integration of two replicated variants (located in or near *BHLHE40-AS1,* and *PLCL2,*) with transcriptomic data suggests a candidate gene in each region with the top GWAS SNP likely influencing risk of SevO through differential expression. Follow-up functional studies for these regions will be prioritized in our future work along with causal modelling to infer genetic liability. Our systematic analysis combining PRS, PheWAS and lifestyle factors further reinforced the limited etiologic heterogeneity between obesity traits, the common downstream sequelae associated with SevO and BMI, and the importance of lifestyle factors in understanding genetic risk for SevO.

## Materials and methods

### Ethics statement

All relevant ethical standards were upheld in the conduct of this research. The current study consisted on secondary analysis of existing data. Any identifiers related to participants, patients, or samples were anonymized and are not known to the research team. This study was deemed Not Human Subjects Research by the Institutional Review Board (IRB) at the University of North Carolina at Chapel Hill (UNC).

Approval was obtained from the appropriate Institutional Review Boards (IRBs) or ethics committees for each participating cohort, and informed consent was secured from all participants. The study adhered to the principles outlined in the Declaration of Helsinki.

Details of the approving IRB or oversight body for each parent study are provided below. All required participant consent forms have been collected and properly archived.

### Study design

Study-specific design, sample quality control and descriptive statistics are provided in S1–S4 Tables. We conducted a two-stage study of SevO traits: 95^th^ percentile [cases defined as the upper 5^th^ percentile and controls as the 5^th^ -50^th^ percentile], Obesity Class III [BMI ≥ 40 kg/m^2^] vs. controls [BMI ≥ 25 kg/m^2^] and Obesity Class IV [BMI ≥ 50 kg/m^2^] vs. controls [BMI ≥ 25 kg/m^2^]. Stage 1 discovery analyses consisted of up to 159,359 individuals (≥18 years to < 70 years) across 11 diverse population-based studies which includes individuals of European (*n = *108,844), African (*n = *23,237), Hispanic (*n = *20,081), East Asian (*n = *4,138), American Indian (*n = *276), Hawaiian (*n = *2,232) and mixed ancestry (*n = *508) backgrounds (S2 Table). Of note, the numbers in the ancestry -combined samples do not match our ancestry-stratified samples because of the inclusion of datasets that did not initially meet our ancestry-stratified criteria (N in cases >20). Discovery meta-analyses were performed in each ancestry group separately and in an all-ancestry combined group for both sex-specific and sex-combined analyses. Given the preponderance of rare variations in our initial discovery, we assessed replication of our novel independent variants that reached GWAS significance threshold (*P = *5x10^-8^) in a stage 2 follow-up samples of 480,897 individuals (≥18 years to < 70 years) across 6 diverse population-based studies which includes individuals of European (*n* = 390,569), African/African American (*n = *62,539), Hispanic (*n = *21,781), South Asian (*n = *4,759) and East Asian (*n = *1,557) ancestries (S2 Table). All studies were approved by local research ethics committees across each institution, and all participants gave informed consent. All methods were performed in accordance with the relevant guidelines and regulations.

### Phenotype definitions

We used 3 different classes of SevO traits defined by BMI (weight (in kg)/height (in m)^2^). Using ancestry specific quantile regression modelling nested within our cohorts, the 95^th^ percentile SevO class was defined as the upper 5th percentile (cases) and 5^th^-50^th^ percentile (controls) of BMI distribution stratified by sex after controlling for age, age^2^ and ancestry (S1–S4 Figs). For clinical obesity classes, cases were defined by BMI ≥ 40 kg/m^2^ for Obesity Class III and BMI ≥ 50 kg/m^2^ for Obesity Class IV. Controls were subjects with BMI ≥ 18.5 kg/m^2^ and < 25 kg/m^2^. A minimum of 20 cases and 20 controls for each study-specific stratum was required for combined analyses, whereas a minimum of 10 cases and 10 controls for each study-specific stratum was required for sex-specific analyses.

### Association analyses

Ancestry and sex specific GWAS, adjusted for age, principal components of ancestry (PCs) 1–10 and study specific covariates, were conducted for the three different classes of SevO traits. Poorly imputed variants (IMPUTE info < 0.4 and/or *R*^2 ^< 3) [67], and those with an effective sample size less than 20 in each stratum (or 10 in each sex-specific stratum) were excluded from association analyses. A centralized quality control procedure implemented in EasyQC [67] was applied to association summary statistics to identify study specific problems. Ancestry and sex combined meta-analysis for each SevO trait was performed in METAL [68] using the fixed-effect inverse variance method based on *β* estimates and standard errors from each study. Variants with a minor allele count [MAC] <50, with a combined sample size of <100, and an inflated heterogeneity test score (Het *I*^*2*^) > 80 (for one study) were excluded from downstream analyses. Similar GWAS, meta-analysis and quality control methods were employed for replication analyses.

### Conditional analysis

We conditioned our significant ten variants of interest on all of the known BMI or obesity variants in the GWAS catalog. We first divided the known variants by background population including European, African, Hispanic/Native American, or Asian. We then pruned these at r2 < 0.5 based on the most aligned reference panel from the 1000 genomes using plink. Then we conditioned our variants of interest on each pruned known list using GCTA-cojo (--cojo-cond flag) and the same reference panel used to prune the respective list [69]. We provide the results of the ten variants of interest based on each known list in S6 Table.

### Gene mapping, functional annotation, and validation of novel variants

Post-GWAS analysis of gene mapping, functional annotation, and tissue expression analysis of prioritized loci in our discovery GWAS was conducted using Functional Mapping and Annotation (FUMA) [70] SNP2GENE function. We identified each locus with an index SNP that met genome-wide significant *P* value threshold of *P* 5x10^-8^ and then included all SNPs surrounding the index SNP ± 250kb on each side. We functionally annotated each locus by considering the index SNP and any SNP in the 500kb region that displayed linkage disequilibrium (LD) *r*^*2*^ < 0.6 with the index SNP using the phase all ancestry 1000G project LD reference panel. If in some loci, two or more SNPs achieved genome-wide significant evidence for association but were independent of one another (*r*^*2*^ < 0.1), we functionally annotate each independent SNP effect. If no genes were present within ±250 kb of the lead variant, the closest gene was selected. We also used PhenoScanner v2 [71], GWAS Catalog [72], and the Integrative Epidemiology Unit (IEU) Open GWAS Project, as well as conducting a comprehensive literature review to evaluate our independent associated variants across all three SevO classes. A variant is considered novel if not previously associated with obesity related traits. Variants that meet these criteria were subsequently assessed in our replication dataset and were considered significant if they were directionally consistent and met our Bonferroni adjusted significance threshold (0.05/*n* independent SNPs) in any SevO class. Independent unreported variants were annotated for predicted pathogenicity by Combined Annotation Dependent Depletion (CADD) scores using CADDv1.3 [73] with a Phred-scale CADD score >10 (top 10%) being deleterious. We utilized online resources such as GeneCards, GWAS Catalog and PubMed to derive potential biological links of genes in proximity of novel signals (±250kb of the index SNP, or the closest gene if no gene was present within ±250 kb) (S16 Table).

### Expression Quantitative Trait Loci (eQTL) and colocalization

We used eQTL summary data from GTEx v8 [34] and eQTLGen Phase 1 release [35], to test for *cis* associations between our novel findings with transcripts within ±1 Mb across obesity related tissues including liver, skeletal muscle, whole blood, brain, and adipose tissues surrounding the transcription start (TSS). Detailed method descriptions can be found in the main GTEx v8 [34] and/or eQTLGen [35] publications. Additionally, we examined *cis* associations between our SevO novel variants with transcripts derived from RNA sequencing (RNAseq) within ±1 Mb in whole blood among 645 Mexican Americans from the County Hispanic Cohort (CCHC) [74]. Significant eQTLs were determined based on Bonferroni adjusted significance threshold (0.05/*n* of transcripts tested for the locus). Colocalization of any validated novel variants in the CCHC cohort was performed using the *coloc* software [75] with a posterior probability >75% supporting colocalization from a single causal variant. Detailed method descriptions on CCHC study population, genotyping and eQTL mapping are provided in Chen *et al*. 2022 [51].

### Binomial concordance analysis

We assessed the rate of concordance using binomial t-test. Between our discovery analysis for Obesity Class III with both meta-analysis and top 714 conditioned BMI associated variants (based on HapMap imputed data from approximately 700,000 European participants identified in Yengo *et al* [17]. Since we were interested in investigating a broader genetic architecture, rather than including only variants with associations surpassing genome-wide significance, we examined variants across three different *P* value thresholds (0.05, 0.005 and 0.0005). We also performed lookups of our unreported independent validated variants (or their LD proxies: R^2^ > 0.8) with SevO traits within this cohort to assess consistency of associations in both the meta-analysis of GIANT and UKBB [17] and the UKBB trans-ancestry meta-analysis of BMI.

### SNP based heritability and genetic correlation between SevO and BMI

Using GWAS summary statistics, we calculated single nucleotide polymorphisms (SNP)-based heritability for SevO traits and the genetic correlation between each trait with BMI using linkage disequilibrium score regression (LDSC) [76,77]. Heritability, ranging from 0 to 1, measures the proportion of variation in a phenotype accounted for by genetic factors. SNP-based genetic correlations, ranging from −1 to +1, measure the extent to which two phenotypes share common genetic variation. Analyses were conducted in the ancestry -combined samples and in European -combined samples only.

### Polygenic risk score (PRS)

Polygenic risk scores (PRS) were generated using GWAS summary statistics from three comparative sets: a) the significant lead independent SNPs from each SevO trait all ancestry GWAS, b) the HapMap 3 SNPs in the all ancestry GWAS of SevO traits from the current study, and c) the HapMap 3 SNPs in the BMI GWAS from the Locke *et al* (2015) paper in the GIANT consortium [19]. Both the UKBB and CCHC test cohorts were not included in the generation of the PRS. We utilized the PRS-continuous shrinkage (CS) method which applies a Bayesian regression framework and places conceptually CS priors on variant effect estimates [78]. Pairwise LD matrices within pre-defined LD blocks [79] (using European LD blocks for LD calculations were calculated using PLINK and converted to HDF5 format [80]). PRS for SevO or BMI was calculated for each ancestral group across all SevO classes. Multivariable logistic regression was used to test the association of PRS SevO or BMI with SevO classes adjusted for age, sex, 10 PCs and ancestry in the UKBB. A partial Nagelkerke *R*^*2*^ was used to estimate the proportion of variance for SevO classes explained by the PRS.

### Investigator-defined phenotypes

For the investigator-defined phenotypes (S22 Table), two analysts independently reviewed each of the 253 UK Biobank phenotype categories to identify those that might include relevant phenotypic information (through December 31, 2022). Categories related to nutrition and diet, those labeled as blob/bulk, and phenotypes assessed in fewer than approximately 10,000 participants were excluded from further consideration. Phenotypes deemed plausibly heritable — excluding variables like type of bread consumed the previous day, occupational codes, or water hardness — underwent manual curation. These were reviewed, renamed where necessary, and grouped into composite outcomes when appropriate (e.g., angina aligned with Rose’s Angina Questionnaire; claudication with the Edinburgh Claudication Questionnaire). As a final quality control step, phenotype distributions were compared against publicly available UK Biobank data (https://biobank.ctsu.ox.ac.uk/crystal/index.cgi) and previously published literature when available.

### Mapping inpatient ICD-10 codes

Inpatient diagnoses recorded using International Classification of Diseases, Tenth Revision (ICD-10) codes were aggregated and mapped to clinically meaningful traits using a phecode-based algorithm [81,82] (S23 Table). Analyses were limited to codes listed in either the primary or secondary diagnosis fields. At the time of analysis, ICD-10 hospitalization data spanning from 1997 to 2021 were available.

### PheWAS using the UKBB

Using the weighted Obesity Class III PRS-BMI we performed a PheWAS of 19 clinical classes of traits in the UKBB for each ancestry (African, European, South Asian, and East Asian). Detailed description of the classes of clinical traits are described in S22 Table. Regression modelling with Obesity Class III PRS-BMI as the independent variable, phecodes as the dependent variables, and age, sex, the first 10 PCs as covariates, were used to identify phenotypic associations. An FDR of 0.05 was applied to account for multiple testing.

### Measures of diagnostic accuracy

Sensitivity, Specificity, Positive Predictive Values (PPV), Negative Predictive Values (NPV) and area under the curve (AUC) was applied in evaluating the effectiveness of SevO PRS only, covariates only and combined SevO PRS and covariates with Obesity Class III across European, African, and South Asian participants from the UKBB and Hispanic participants from the CCHC cohort.

### PRS deciles and assessment of lifestyle factors

We stratified UKBB participants by their Class III SevO PRS-BMI according to deciles (1–10) with high PRS-BMI comprising >90^th^ percentile (decile 10) and low PRS-BMI the ≤ 10^th^ percentile (decile 1). Normal weight (≤25 kg/m^2^), overweight (>25 kg/m^2 ^≤ 30 kg/m^2^) obesity (>30 kg/m^2 ^≤ 40 kg/m^2^) and SevO Class 3 (>40 kg/m^2^) was determined within each decile for ancestry -stratified and -combined groups. We explored the predictive power of our PRS-BMI on severe obesity (≥ 40 kg/m^2^) between our > 90^th^ percentile (*n =* 42,844) and ≤10^th^ percentile (*n =* 42,856) in the ancestry -combined samples within UKBB. We then assessed whether patterns of lifestyle factor associations, including physical activity, dietary patterns, alcohol/smoke servings, sleep behaviors and self-reported “body size” at age 10, were differentially associated in individuals across the PRS-BMI deciles. Those with a PRS in the ≥ 90^th^ percentile and a BMI < 25 kg/m^2^ were labeled the “Resilient group”. Lifestyle behavior regression modelling was compared between our “Resilient group” and those with 1) PRS and SevO; 2) >90^th^ percentile for PRS and SevO Class III; 3) < 10^th^ percentile for PRS and SevO and 4) <10^th^ percentile and BMI < 25 kg/m^2^.

## Supporting information

S1 Table Study design, genotype and imputation quality control steps, and analysis for each contributing study. (XLSX)

S2 Table Phenotype descriptives by study, sex strata, and obesity definition. (XLSX)

S3 Table Sample size distributions of severe obesity classes by self-identified ethnic or ancestry background group (discovery population). (XLSX)

S4 Table Sample size distributions of severe obesity classes by self-identified ethnic or ancestry background group (replication population). (XLSX)

S5 Table Discovery and Replication of novel independent variants in all populations. (XLSX)

S6 Table Conditional analyses of potentially novel independent variants by known variants by ancestry. (XLSX)

S7 Table Sex differences among potentially novel independent variants and any known loci with genome-wide significance among all populations. (XLSX)

S8 Table GWAS summary statistics for potentially novel independent variants in the Discovery meta-analysis for the 95% obesity trait by self-identified background. (XLSX)

S9 Table GWAS summary statistics for potentially novel independent variants in the Discovery meta-analysis for the class 3 obesity trait by self-identified background. (XLSX)

S10 Table GWAS summary statistics for potentially novel independent variants in the Discovery meta-analysis for the class 4 obesity trait by self-identified background. (XLSX)

S11 Table GWAS summary statistics for potentially novel independent variants in the replication meta-analysis for the 95% obesity trait by self-identified background. (XLSX)

S12 Table GWAS summary statistics for potentially novel independent variants in the replication meta-analysis for the class 3 obesity trait by self-identified background. (XLSX)

S13 Table GWAS summary statistics for potentially novel independent variants in the replication meta-analysis for the class 4 obesity trait by self-identified background. (XLSX)

S14 Table Expression quantitative trait loci (eQTL) measurement for validated novel severe obesity SNPs in GTEx. We restricted analyses to adiposity associated tissues (whole blood, skeletal muscle, adipose tissue, liver, brain).
(XLSX)

S15 Table Expression quantitative trait (eQTL) measured for validated severe obesity SNPs in whole blood (transcripts expression levels measured in Cameron County Hispanic Cohort [CCHC] whole blood tissue). (XLSX)

S16 Table Literature review on functional role of genes (±250kb) or genes closest to the index variant in our validated signals. (XLSX)

S17 Table Functional scoring of severe obesity validated SNPs across each strata. Highlighted rows suggest SNPS with CADD score >10 which suggest likely to be deleterious.
(XLSX)

S18 Table Binomial concordance rates between all ancestry class 3 obesity SNP versus BMI GWAS meta-analysis by Yengo + Locke (Yengo et al., 2018). (XLSX)

S19 Table Binomial concordance rates between all ancestry class 3 obesity SNP versus BMI GWAS conditional analysis by Yengo + Locke (Yengo et al., 2018). (XLSX)

S20 Table Polygenic scores based on PRS-CS and significant SNP methods using top fine-mapped independent variants by trait. (XLSX)

S21 Table Correlations scores between BMI PRS and Obesity Class 3 PRS across unrelated individuals. (XLSX)

S22 Table Description of investigator-measured continuous and categorical phenotypes assessed in UK Biobank participants. (XLSX)

S23 Table Description of inpatient phenotypes and phecodes-ICD-10 codes mapped in UK Biobank participants. (XLSX)

S24 Table Summary total FDR adjusted significant phenome-wide associations by trait category and population. (XLSX)

S25 Table Phenome-wide association test in self-identified European ancestry, with PRS-CS for severe obesity as predictors. (XLSX)

S26 Table Phenome-wide association test in self-identified East Asians ancestry, with PRS-CS for severe obesity as predictors. (XLSX)

S27 Table Phenome-wide association test in self-identified African ancestry, with PRS-CS for severe obesity as predictors. (XLSX)

S28 Table Phenome-wide association test in self-identified South Asian ancestry, with PRS-CS for severe obesity as predictors. (XLSX)

S29 Table Sample sizes by PRS decile and BMI category and ancestry background in the UKBB. (XLSX)

S30 Table BMI distributions by self-identified ancestry groups comparing those in PRS > 90^th^ percentile versus individuals in <10^th^ percentile category. (XLSX)

S31 Table Behavioral comparisons between sample groups defined by PRS upper and lower deciles and BMI categories. (XLSX)

S32 Table Heritability and genetic correlation of each Severe Obesity trait (SevO) with BMI in the all and European ancestry cohorts. (XLSX)

S33 Table Predictive value of the SevO PRS status for Obesity Class 3 across diverse ancestries. (XLSX)

S1 Fig Women, standard cut-offs versus by quantile regression. Jagged lines for %tile cut-offs are because all races are shown together.
(DOCX)

S2 Fig Men, standard cut-offs versus by quantile regression. Jagged lines for %tile cut-offs are because all races are shown together.
(DOCX)

S3 Fig Women, quantile regression by race/ethnicity. (DOCX)

S4 Fig Men, quantile regression by race/ethnicity. (DOCX)

S5 Fig QQ (upper) and Manhattan (lower) plots for All-ancestry sex combined 95^th^ percentile severely obese vs 5^th^ to 50^th^ percentile normal weight controls. Black dots in QQ plot denote expected vs observed -log 10 p-values for association without controlling for known obesity loci, while dark orange dots denote expected vs observed after controlling for known obesity loci. Blue dots in Manhattan plot denote known signals and red dots suggest possible novel signals.
(DOCX)

S6 Fig QQ (upper) and Manhattan (lower) plots for All-ancestry female 95^th^ percentile severely obese vs 5^th^ to 50^th^ percentile normal weight controls. Black dots in QQ plot denote expected vs observed -log 10 p-values for association without controlling for known obesity loci, while dark orange dots denote expected vs observed after controlling for known obesity loci. Blue dots in Manhattan plot denote known signals and red dots suggest possible novel signals.
(DOCX)

S7 Fig QQ (upper) and Manhattan (lower) plots for All-ancestry male 95^th^ percentile severely obese vs 5^th^ to 50^th^ percentile normal weight controls. Black dots in QQ plot denote expected vs observed -log 10 p-values for association without controlling for known obesity loci, while dark orange dots denote expected vs observed after controlling for known obesity loci. Blue dots in Manhattan plot denote known signals and red dots suggest possible novel signals.
(DOCX)

S8 Fig QQ (upper) and Manhattan (lower) plots for All-ancestry sex combined class III (BMI ≥ 40 kg/m^2^) vs normal weight (18 kg/m^2^ ≤ BMI < 25 kg/m^2^) controls. Black dots in QQ plot denote expected vs observed -log 10 p-values for association without controlling for known obesity loci, while dark orange dots denote expected vs observed after controlling for known obesity loci. Blue dots in Manhattan plot denote known signals and red dots suggest possible novel signals.
(DOCX)

S9 Fig QQ (upper) and Manhattan (lower) plots for All-ancestry female class III (BMI ≥ 40 kg/m^2^) vs normal weight (18 kg/m^2^ ≤ BMI < 25 kg/m^2^) controls. Black dots in QQ plot denote expected vs observed -log 10 p-values for association without controlling for known obesity loci, while dark orange dots denote expected vs observed after controlling for known obesity loci. Blue dots in Manhattan plot denote known signals and red dots suggest possible novel signals.
(DOCX)

S10 Fig QQ (upper) and Manhattan (lower) plots for All-ancestry male class III (BMI ≥ 40 kg/m^2^) vs normal weight (18 kg/m^2^ ≤ BMI < 25 kg/m^2^) controls. Black dots in QQ plot denote expected vs observed -log 10 p-values for association without controlling for known obesity loci, while dark orange dots denote expected vs observed after controlling for known obesity loci. Blue dots in Manhattan plot denote known signals and red dots suggest possible novel signals.
(DOCX)

S11 Fig QQ (upper) and Manhattan (lower) plots for All-ancestry sex combined class IV (BMI ≥ 50 kg/m^2^) vs normal weight (18 kg/m^2^ ≤ BMI < 25 kg/m^2^) controls. Black dots in QQ plot denote expected vs observed -log 10 p-values for association without controlling for known obesity loci, while dark orange dots denote expected vs observed after controlling for known obesity loci. Blue dots in Manhattan plot denote known signals and red dots suggest possible novel signals.
(DOCX)

S12 Fig QQ (upper) and Manhattan (upper) plots for All-ancestry female class 4 (BMI ≥ 50 kg/m^2^) vs normal weight (18 kg/m^2^ ≤ BMI < 25 kg/m^2^) controls. Black dots in QQ plot denote expected vs observed -log 10 p-values for association without controlling for known obesity loci, while dark orange dots denote expected vs observed after controlling for known obesity loci. Blue dots in Manhattan plot denote known signals and red dots suggest possible novel signals.
(DOCX)

S13 Fig QQ (upper) and Manhattan (lower) plots for All-ancestry female class 4 (BMI ≥ 50 kg/m^2^) vs normal weight (18 kg/m^2^ ≤ BMI < 25 kg/m^2^) controls. Black dots in QQ plot denote expected vs observed -log 10 p-values for association without controlling for known obesity loci, while dark orange dots denote expected vs observed after controlling for known obesity loci. Blue dots in Manhattan plot denote known signals and red dots suggest possible novel signals.
(DOCX)

S14 Fig Phenome-wide association of severe Obesity Class III polygenic risk score with clinical traits in self-identified East Asian, African, and South Asian ancestry groups. (DOCX)

S15 Fig BMI distributions by self-identified ancestry groups comparing those in PRS > 90^th^ percentile versus individuals in <10^th^ percentile category. African and East Asian ancestry groups in the lower 10^th^ percentile group show noticeably higher and lower BMI distributions compared to other ancestries respectively. East Asians show lower average BMI distributions among those in high 90^th^ percentile category, but Africans demonstrate similar BMI patterns to other ancestries. (Note: AFR (African ancestry), EAS (East Asian ancestry), EUR (European ancestry), and SAS (South Asian ancestry)).
(DOCX)

S16 Fig Box plots of BMI across PRS deciles in the UKBB by ancestry background. Upward, linear association can be observed between PRS deciles and BMI in all populations. (Note: AFR (African ancestry), EAS (East Asian ancestry), EUR (European ancestry), and SAS (South Asian ancestry)).
(DOCX)

S1 Data Includes the Internal Review Board or Ethics Committee details for each study that participated. (DOCX)

S2 Data Includes details about study design for each of the participating studies. (DOCX)
