# Supplementary material for: Genome-wide association study provides novel insight into the genetic architecture of severe obesity
Source: PLoS Genet. 2025 Sep 12;21(9):e1011842. doi: 10.1371/journal.pgen.1011842 (PMC12443252; doi:10.1371/journal.pgen.1011842)

**Supplementary Figure 2.** Men, standard cut-offs versus by quantile regression. Jagged lines for %tile cut-offs are because all races are shown together.


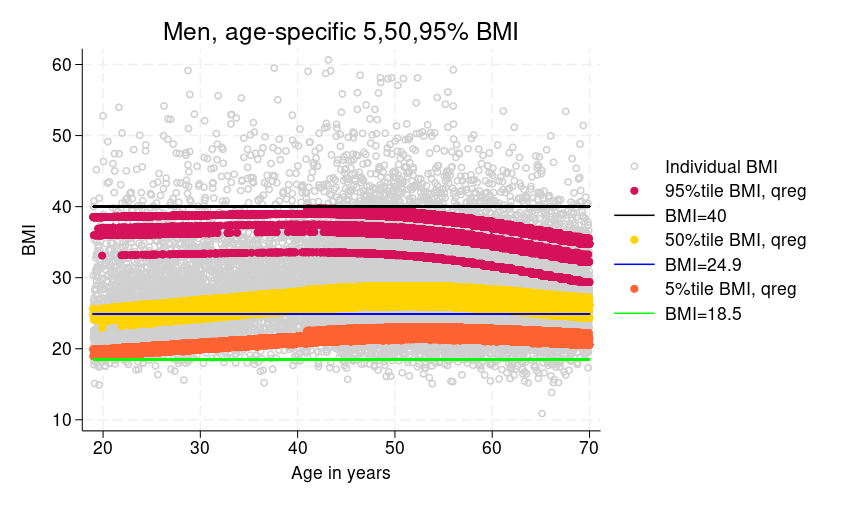

Supplement: S2 Fig — Jagged lines for %tile cut-offs are because all races are shown together. (DOCX) [file pgen.1011842.s035.docx]
