# Supplementary figures and images for: Genome-wide association study provides novel insight into the genetic architecture of severe obesity
Source: PLoS Genet. 2025 Sep 12;21(9):e1011842. doi: 10.1371/journal.pgen.1011842 (PMC12443252; doi:10.1371/journal.pgen.1011842)

**Supplementary Figure 3.** Women, quantile regression by race/ethnicity.


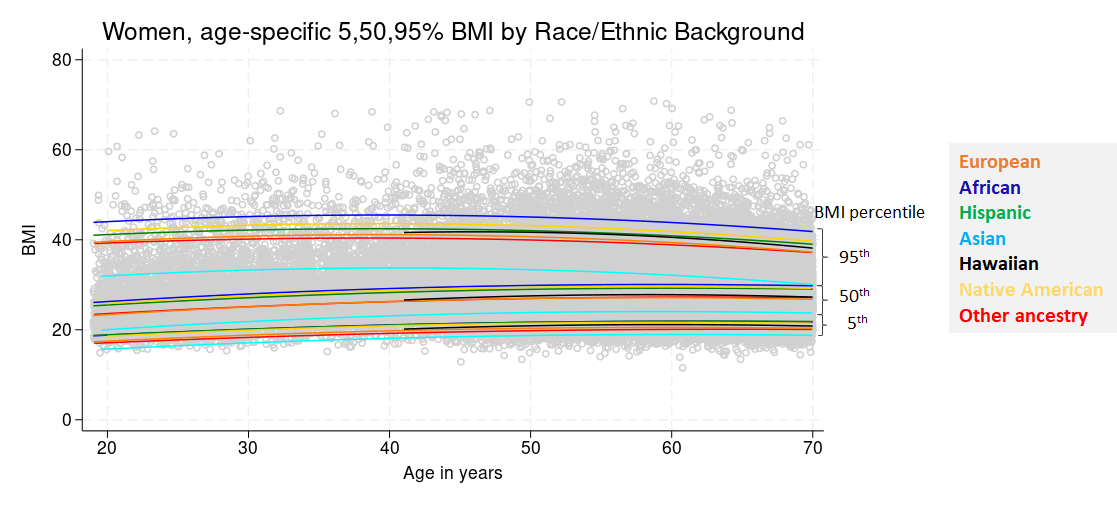

Supplement: S3 Fig — (DOCX) [file pgen.1011842.s036.docx]

**Supplementary Figure 4.** Men, quantile regression by race/ethnicity.


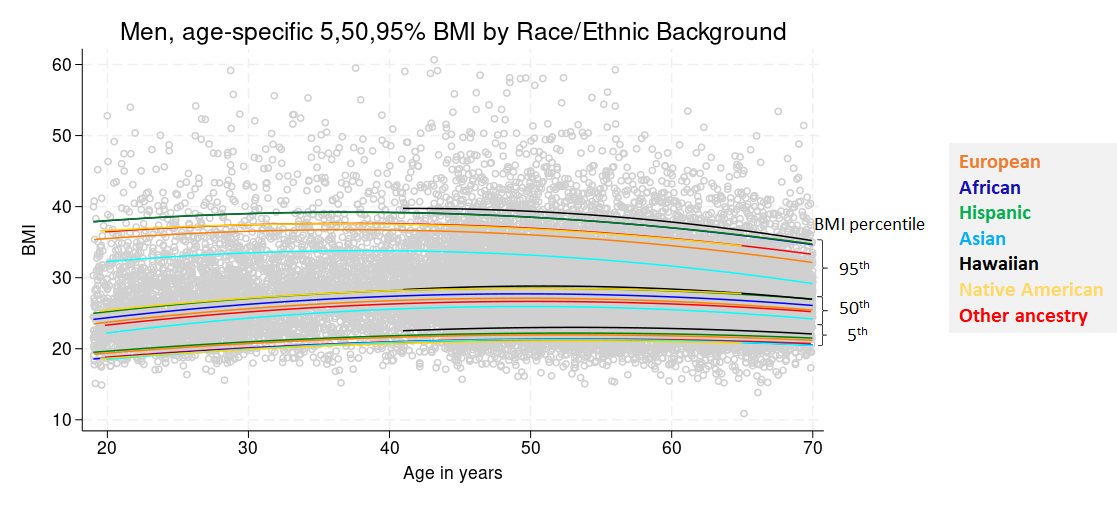

Supplement: S4 Fig — (DOCX) [file pgen.1011842.s037.docx]
