# Supplementary material for: Genome-wide association study provides novel insight into the genetic architecture of severe obesity
Source: PLoS Genet. 2025 Sep 12;21(9):e1011842. doi: 10.1371/journal.pgen.1011842 (PMC12443252; doi:10.1371/journal.pgen.1011842)

**Supplementary Figure 7.** QQ (upper) and Manhattan (lower) plots for All-ancestry male 95th percentile severely obese vs 5th to 50th percentile normal weight controls. Black dots in QQ plot denote expected vs observed -log 10 p-values for association without controlling for known obesity loci, while dark orange dots denote expected vs observed after controlling for known obesity loci. Blue dots in Manhattan plot denote known signals and red dots suggest possible novel signals.


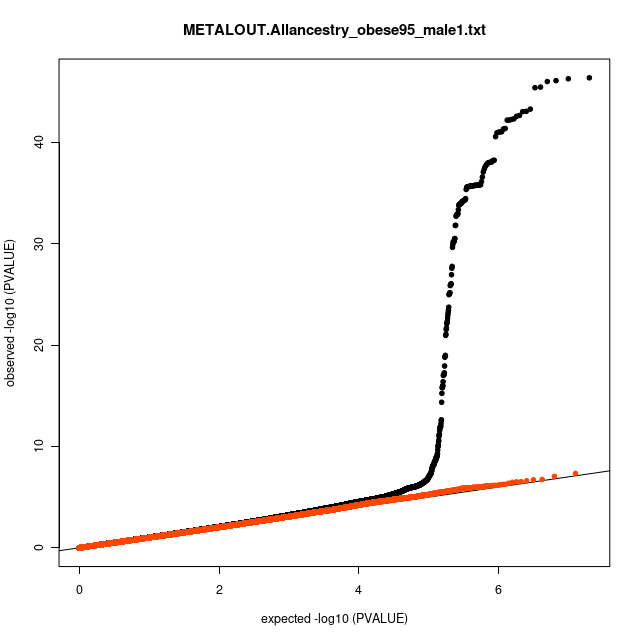


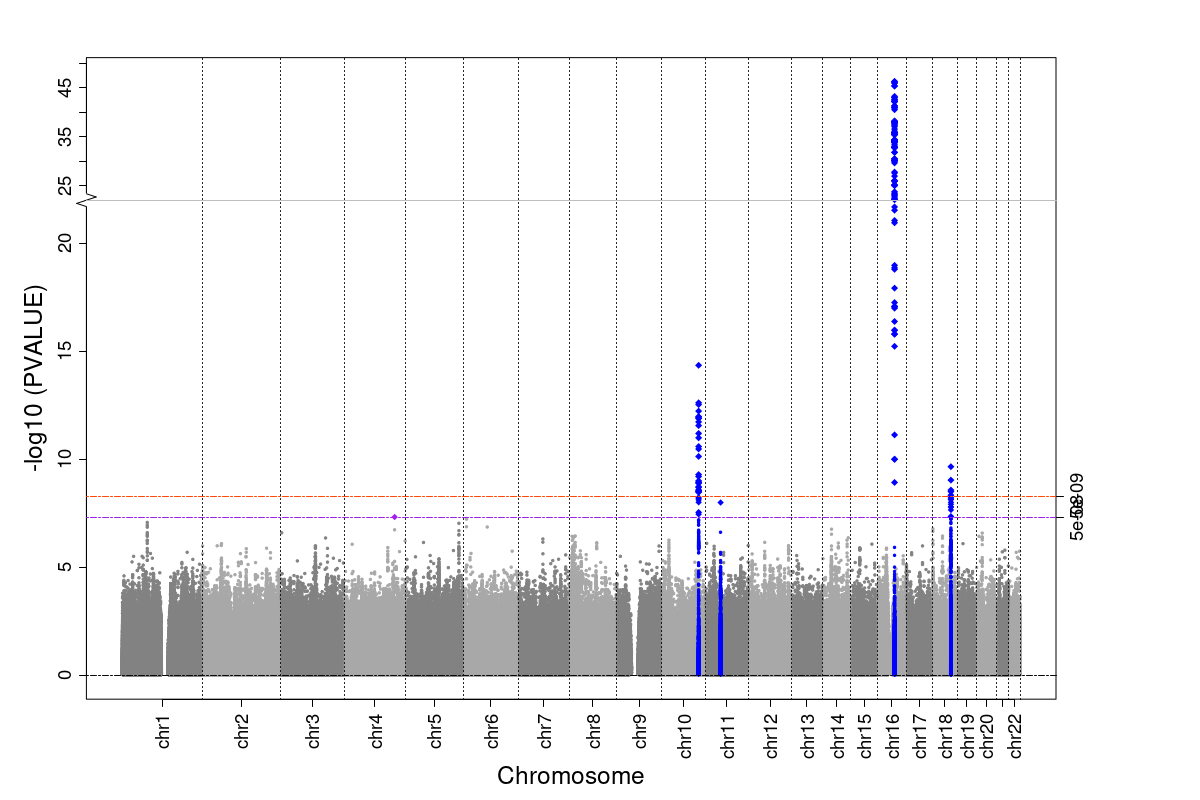

Supplement: S7 Fig — Black dots in QQ plot denote expected vs observed -log 10 p-values for association without controlling for known obesity loci, while dark orange dots denote expected vs observed after controlling for known obesity loci. Blue dots in Manhattan plot denote known signals and red dots suggest possible novel signals. (DOCX) [file pgen.1011842.s040.docx]
