# Supplementary material for: Genome-wide association study provides novel insight into the genetic architecture of severe obesity
Source: PLoS Genet. 2025 Sep 12;21(9):e1011842. doi: 10.1371/journal.pgen.1011842 (PMC12443252; doi:10.1371/journal.pgen.1011842)

**Supplementary Figure 14.** Phenome-wide association of severe Obesity Class III polygenic risk score with clinical traits in self-identified East Asian, African, and South Asian ancestry groups.


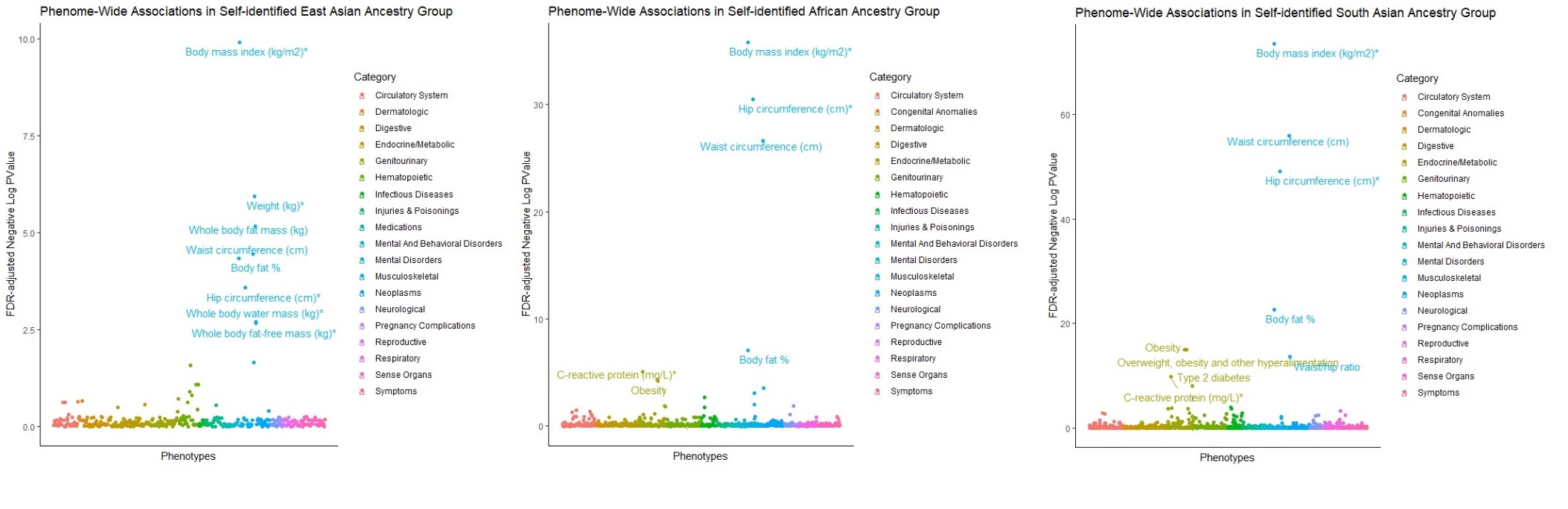

Supplement: S14 Fig — (DOCX) [file pgen.1011842.s047.docx]
