# Supplementary material for: Genome-wide association study provides novel insight into the genetic architecture of severe obesity
Source: PLoS Genet. 2025 Sep 12;21(9):e1011842. doi: 10.1371/journal.pgen.1011842 (PMC12443252; doi:10.1371/journal.pgen.1011842)

**Supplementary Figure 15.** BMI distributions by self-identified ancestry groups comparing those in PRS>90^th^ percentile versus individuals in <10^th^ percentile category. African and East Asian ancestry groups in the lower 10^th^ percentile group show noticeably higher and lower BMI distributions compared to other ancestries respectively. East Asians show lower average BMI distributions among those in high 90^th^ percentile category, but Africans demonstrate similar BMI patterns to other ancestries. (Note: AFR (African ancestry), EAS (East Asian ancestry), EUR (European ancestry), and SAS (South Asian ancestry))


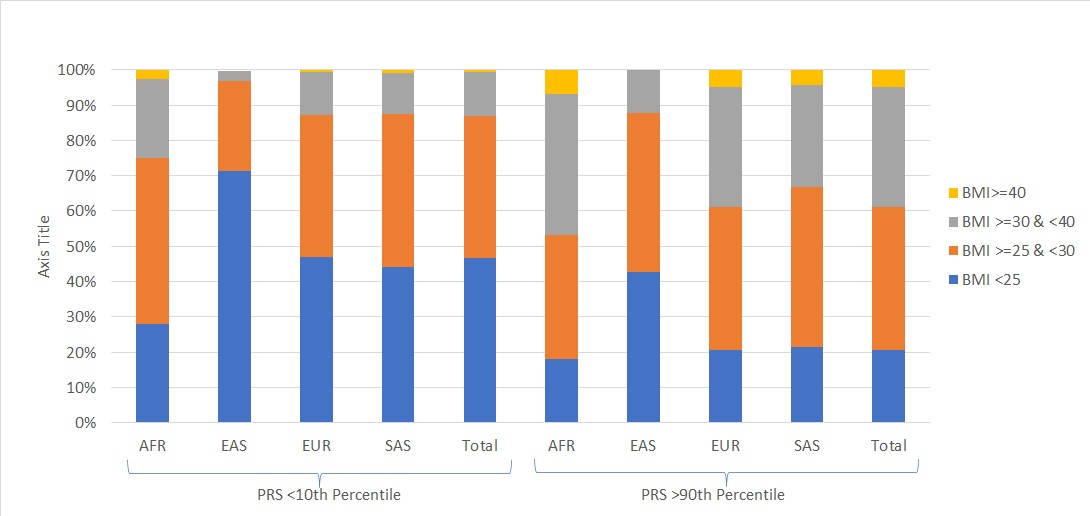

Supplement: S15 Fig — African and East Asian ancestry groups in the lower 10th percentile group show noticeably higher and lower BMI distributions compared to other ancestries respectively. East Asians show lower average BMI distributions among those in high 90th percentile category, but Africans demonstrate similar BMI patterns to other ancestries. (Note: AFR (African ancestry), EAS (East Asian ancestry), EUR (European ancestry), and SAS (South Asian ancestry)). (DOCX) [file pgen.1011842.s048.docx]
