# Supplementary material for: Genome-wide association study provides novel insight into the genetic architecture of severe obesity
Source: PLoS Genet. 2025 Sep 12;21(9):e1011842. doi: 10.1371/journal.pgen.1011842 (PMC12443252; doi:10.1371/journal.pgen.1011842)

**Supplementary Figure 16.** Box plots of BMI across PRS deciles in the UKBB by ancestry background. Upward, linear association can be observed between PRS deciles and BMI in all populations. (Note: AFR (African ancestry), EAS (East Asian ancestry), EUR (European ancestry), and SAS (South Asian ancestry))


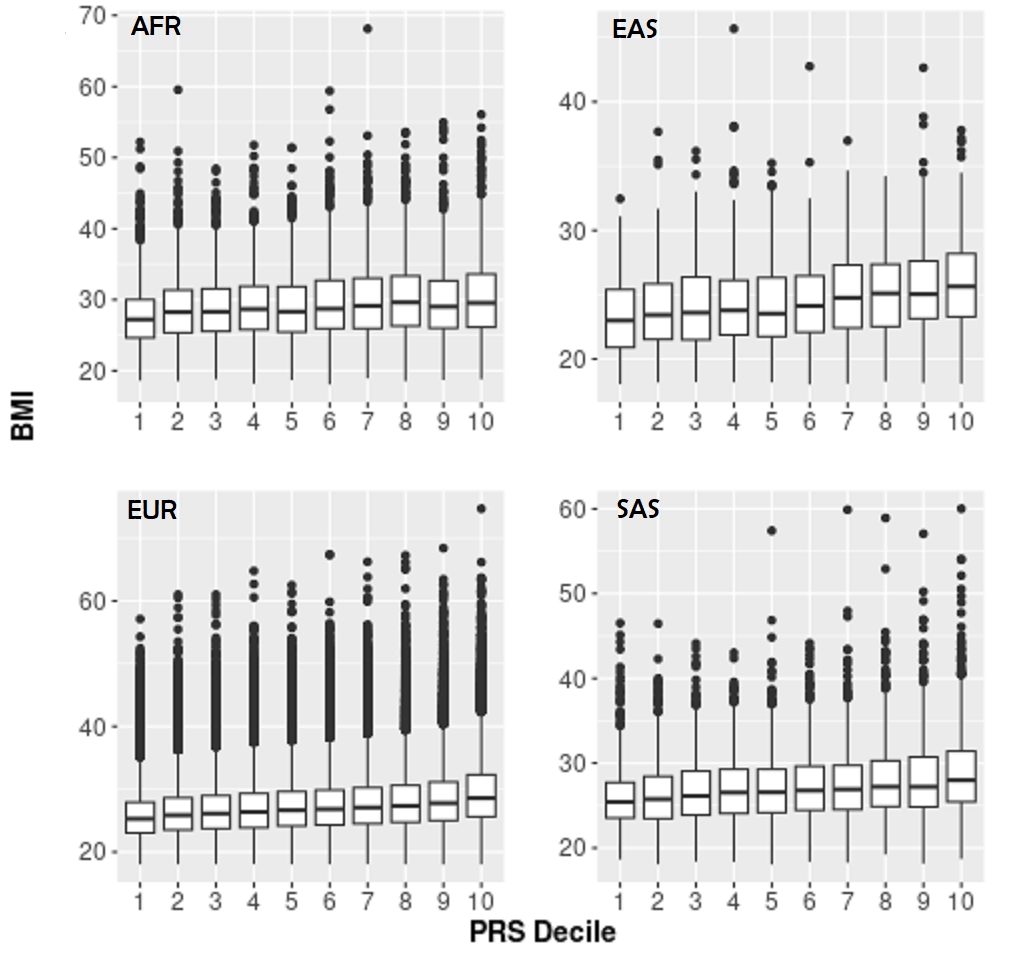

Supplement: S16 Fig — Upward, linear association can be observed between PRS deciles and BMI in all populations. (Note: AFR (African ancestry), EAS (East Asian ancestry), EUR (European ancestry), and SAS (South Asian ancestry)). (DOCX) [file pgen.1011842.s049.docx]
