# Supplementary material for: Genome-wide association study provides novel insight into the genetic architecture of severe obesity
Source: PLoS Genet. 2025 Sep 12;21(9):e1011842. doi: 10.1371/journal.pgen.1011842 (PMC12443252; doi:10.1371/journal.pgen.1011842)
Supplement: S1 Data — (DOCX) [file pgen.1011842.s050.docx]

**Internal Review Board and Ethics Committee by each study in the project**

1. **Discovery studies**

**The Population Architecture using Genomics and Epidemiology (PAGE) Consortium**

*Atherosclerosis Risk in Communities Study (ARIC)*

Atherosclerosis Risk in Communities Study (ARIC): The ARIC study is approved by the Collaborative Studies Coordinating Center with the IRB at UNC, and each of the original field centers, Wake Forest University, University of Mississippi Medical Center, University of Minnesota, and Johns Hopkins University..

*The BioMe Biobank Program (BioMe)*

The BioMe Biobank Program (BioMe) : This research study was reviewed and approved by the ethics review board of the Program for the Protection of Human Subjects (PPHS) of Mount Sinai School of Medicine under project # HSD09-00030. The Mount Sinai Biobank Project (IRB # 07-0529 0001 02 ME) is an IRB-approved research protocol with IRB-approved informed consent forms. All study participants provided written informed consent.

*The Coronary Artery Risk Development in Young Adults Study (CARDIA)*

The Coronary Artery Risk Development in Young Adults Study (CARDIA): All participants provided written informed consent, with institutional review board approval at each field center (University of Alabama at Birmingham, Northwestern University, University of Minnesota, and Kaiser Permanente).

*The Hispanic Community Health Study/Study of Latinos (HCHS/SOL)*

This study was approved by the institutional review boards (IRBs) at each field center, where all participants gave written informed consent, and by the Non-Biomedical IRB at the University of North Carolina at Chapel Hill, to the HCHS/SOL Data Coordinating Center. All IRBs approving the study are: Non-Biomedical IRB at the University of North Carolina at Chapel Hill. Chapel Hill, NC; Einstein IRB at the Albert Einstein College of Medicine of Yeshiva University. Bronx, NY; IRB at Office for the Protection of Research Subjects (OPRS), University of Illinois at Chicago. Chicago, IL; Human Subject Research Office, University of Miami. Miami, FL; Institutional Review Board of San Diego State University. San Diego, CA. The IRB numbers at the University of North Carolina for the Hispanic Community Health Study/Study of Latinos (HCHS/SOL) include 0185 (2023), 0639 (2020), 0323B (2019), and 0319 (2018).

*The Multiethnic Cohort Study (MEC)*

The Multiethnic Cohort Study (MEC): Receipt of a completed, mailed baseline questionnaire was considered implicit consent to participate in the MEC by the institutional review boards (IRBs) of the University of Hawaii and the University of Southern California.

*The Women’s Health Initiative Study (WHI)*

The WHI project was reviewed and approved by the Fred Hutchinson Cancer Research Center (Fred Hutch) IRB in accordance with the U.S. Department of Health and Human Services regulations at 45 CFR 46 (approval number: IR# 3467-EXT). Participants provided written informed consent to participate. Additional consent to review medical records was obtained through signed written consent. Fred Hutch has an approved FWA on file with the Office for Human Research Protections (OHRP) under assurance number 0001920.

**Mexico City Studies (MC1 & MC2)**

Mexico City Studies (MC1 & MC2): Mexico City-sample 1 and 2 studies, informed consent was obtained from each participant, and the ethical research board (Comite Local de Investigacion) of the Medical Center Siglo XXI approved the research, and the methods were carried out in accordance with the approved guidelines. The Ethics Review Office at the University of Toronto also approved this study.

**Framingham Heart Study (FHS)**

The Framingham Heart Study was approved by the Institutional Review Board of the Boston University Medical Center. All study participants provided written informed consent.

**Netherlands Epidemiology of Obesity (NEO)**

The NEO study was approved by the medical ethical committee of the Leiden University Medical Center (LUMC).

**BioVU**

The Vanderbilt Institutional Review Board (IRB) reviewed the initial project plan and agreed that it met the criteria to be designated as nonhuman subjects research. However, given the anticipated scale of the project and its potential impact on the community, the IRB advised additional safeguards. These included ongoing institutional and IRB oversight; evaluation by the Medical Centers Ethics committee; and establishment of Ethics, Scientific, and Community Advisory Boards.

**MyCode/DiscovEHR**

The MyCode Community Initiative parent study was approved by the Geisinger IRB (Study # 2006-0258). The MyCode Governing Board reviews and approves all uses of MyCode samples and data. Additionally, the Geisinger IRB reviewed this study and determined the study did not involve human subjects as defined in 45 CFR 46. 102(f); and therefore was not subject to additional oversight by the IRB (Study #2017-158).

**B. Replication or Follow-up analyses**

**Million Veteran Program (MVP)**

MVP has received ethical and study protocol approval by the Veterans Affairs Central Institutional Review Board in accordance with the principles outlined in the Declaration of Helsinki.

**REasons for Geographic and Racial Differences in Stroke (REGARDS)**

The organization of REGARDS comprises an Operations Center and the Survey Research Unit (SRU) at the University of Alabama at Birmingham, a Central Laboratory at the University of Vermont, an Electrocardiogram (ECG) Reading Center at Wake Forest University, an in-home exam component provided by Examination Management Services, Inc. (EMSI), and a medical monitoring and stroke adjudication center at Alabama Neurological Institute, Inc. An Executive Committee comprising the principal investigator of each study center and a National Institute of Neurological Disorders and Stroke representative assists the principal investigator at the University of Alabama at Birmingham in the scientific leadership of the study. Study methods were reviewed and approved by all involved institutional review boards, as well as an external observational study monitoring board appointed by the funding agency.

**Genetic Studies of Atherosclerosis Risk (GeneSTAR)**

All participants provided written informed consent and the study was approved by the Johns Hopkins Medicine Institutional Review Board.

**Health and Retirement Study (HRS)**

HRS was approved by the by the University of Michigan Health Sciences/Behavioral Sciences IRB for the Survey Research Center, Institute for Social Research, University of Michigan.

**Genetic Epidemiology Network of Arteriopathy (GENOA)**

The study protocol was approved by the Human Studies Review Boards of the University of Mississippi Medical Center, the Mayo Clinic, and the University of Texas. Informed consent was obtained from all study participants. Written informed consent was obtained from all subjects and approval was granted by participating IRBs at the University of Michigan, University of Mississippi Medical Center, and Mayo Clinic.

**UK Biobank (UKBB)**

Ethics approval for the UK Biobank study was obtained from the North West Centre for Research Ethics Committee (11/NW/0382).

**Cameron County Hispanic Cohort (CCHC)**

The Cameron County Hispanic Cohort (CCHC) study was approved by the IRB of the University of Texas Health Science Center-Houston. All participants provided written consent at each study visit.
